# Mitigating Future Respiratory Virus Pandemics: New Threats and Approaches to Consider

**DOI:** 10.3390/v13040637

**Published:** 2021-04-08

**Authors:** Gregory C. Gray, Emily R. Robie, Caleb J. Studstill, Charles L. Nunn

**Affiliations:** 1 Division of Infectious Diseases, Duke University School of Medicine, Durham, NC 27710, USA; emily.robie@duke.edu (E.R.R.); calebjstudstill@gmail.com (C.J.S.); 2 Duke Global Health Institute, Duke University, Durham, NC 27710, USA; clnunn@duke.edu; 3 Emerging Infectious Disease Program, Duke-NUS Medical School, Singapore 169856, Singapore; 4 Global Health Center, Duke Kunshan University, Kunshan 215316, China; 5 Department of Evolutionary Anthropology, Duke University, Durham, NC 27708, USA

**Keywords:** pathogen discovery, molecular detection, respiratory viruses, emerging viruses

## Abstract

Despite many recent efforts to predict and control emerging infectious disease threats to humans, we failed to anticipate the zoonotic viruses which led to pandemics in 2009 and 2020. The morbidity, mortality, and economic costs of these pandemics have been staggering. We desperately need a more targeted, cost-efficient, and sustainable strategy to detect and mitigate future zoonotic respiratory virus threats. Evidence suggests that the transition from an animal virus to a human pathogen is incremental and requires a considerable number of spillover events and considerable time before a pandemic variant emerges. This evolutionary view argues for the refocusing of public health resources on novel respiratory virus surveillance at human–animal interfaces in geographical hotspots for emerging infectious diseases. Where human–animal interface surveillance is not possible, a secondary high-yield, cost-efficient strategy is to conduct novel respiratory virus surveillance among pneumonia patients in these same hotspots. When novel pathogens are discovered, they must be quickly assessed for their human risk and, if indicated, mitigation strategies initiated. In this review, we discuss the most common respiratory virus threats, current efforts at early emerging pathogen detection, and propose and defend new molecular pathogen discovery strategies with the goal of preempting future pandemics.

## 1. Introduction

In this report, we seek to review the impact that respiratory viruses have had upon mankind, discuss current efforts regarding their control, and propose new strategies to detect emerging respiratory virus threats and mitigate them before they become widespread. The goal in proposing new strategies is to identify methods that will be widely embraced, preemptive, and sustainable, rather than our current practice of being surprised by new threats. The latter forces countries to invest intensive and expensive response efforts towards respiratory virus control, while enduring significant morbidity.

In recent decades, the world has experienced numerous epidemics of emerging or reemerging respiratory viruses. These viruses have been responsible for much morbidity and mortality (Table 1), in addition to pandemics in 2009 and 2020. While these viruses come from five different viral families (*Adenoviridae*, *Coronaviridae*, *Orthomyxoviridae*, *Picornaviridae,* and *Pneumoviridae*), they have two things in common: many are RNA viruses, and most are zoonotic. A review of these epidemics is necessary for planning better future mitigation strategies.

### 1.1. Human Metapneumovirus (hMPV)

hMPV was first discovered in 2001 in the Netherlands, yet it is thought to have been in circulation for at least 50 years beforehand [11]. Retrospective specimen analyses in the USA have found evidence of hMPV as far back as 1982 [12]. Phylogenetic analyses hypothesize divergence from an avian predecessor to have occurred around 200 years ago [13]. Serological studies show that, worldwide, nearly all children develop antibodies to this commonly circulating virus by age five, accounting for 6–40% of acute respiratory infections in this age group [14]. Surveillance efforts are primarily targeted towards young children, but reinfection throughout adulthood is common, with elderly and immunocompromised individuals at heightened risk of severe outcomes [14]. The virus is thought to have spilled over between avian species and man [15,16] as well as between nonhuman primates and man [15,16,17]. This RNA virus belongs to the *Pneumoviridae* family (new classification as of 2016, formerly a subfamily within the *Paramyxoviridae* family).

### 1.2. Rhinovirus Group C (HRV-C)

HRV-C was identified in 2004, following an outbreak of influenza-like illness in New York State [18]. In limited surveillance studies, HRV-C has since been identified as the most prevalent circulating strain of rhinovirus in the fall and winter seasons of multiple countries [2]. HRV-C is associated with more severe respiratory disease than rhinoviruses A and B, and has been connected to asthma, bronchitis, wheezing, and pneumonia [2]. The virus has crossed species from man to nonhuman primates [19]. This RNA virus belongs to the *Picornaviridae* family.

### 1.3. Enterovirus A71 (EV-A71)

EV-A71 infections are recognized as the cause of hand, foot, and mouth disease and various neurological conditions, especially among children under five years. The virus was first isolated from an encephalitic patient in California in 1969, and between 1972 and 1990 was associated with additional outbreaks across six countries [3]. EV-A71 became endemic in the Asian-Pacific region in the 1990s, with major outbreaks occurring every three to four years, which affected thousands of children. Surveillance is sparse, yet the virus is estimated to be responsible for at least 12.8 M infections and 3747 deaths in Asia alone [3]. Large outbreaks have primarily been limited to the Asian-Pacific region; however, the virus still has a global presence, as indicated by small-scale flare-ups, such as the 119 cases reported in Australia in 2012 [3] and 34 cases seen in Colorado in 2018 [20]. While some enteroviruses are recognized to be cross-species [21], this RNA virus is not thought to be zoonotic. It belongs to the *Picornaviridae* family.

### 1.4. Severe Acute Respiratory Syndrome Coronavirus (SARS-CoV)

SARS-CoV emerged in China in 2003, and rapidly spread to 29 countries before it was halted, with heroic public health and hospital infection control measures. Though no cases of SARS-CoV infection have been identified since 2004, it is recognized to have infected more than 8000 people and caused 774 deaths during the short time the virus was circulating [4]. The virus is thought have originated in bats and moved to man via one or more intermediate animal hosts [22]. This RNA virus belongs to the *Coronaviridae* family.

### 1.5. Human Adenovirus 14 Strain (HAdV14)

HAdV14 reemerged in the United States in 2006, causing at least 750 illnesses and 13 deaths [5]. As with other such viruses, surveillance for this “killer cold virus” is sparse, however, available data suggest that it has spread to at least Ireland, Canada and China, and still circulates today. While some adenoviruses have jumped species [23], this adenovirus is not thought to be zoonotic. This DNA virus belongs to the *Adenoviridae* family.

### 1.6. 2009 H1N1 Influenza A Pandemic (H1N1pdm09) Virus 

The H1N1pdm09 virus emerged in Mexico in 2009, and quickly spread throughout the world, causing an estimated 60.8 million illnesses and at least 12,469 deaths from 2009–2010 in the United States alone [6,24]. This swine-like influenza virus, which continues to circulate today in both humans and pigs, served as a catalyst for more comprehensive influenza A virus surveillance strategies. This RNA virus belongs to the *Orthomyxoviridae* family.

### 1.7. Middle East Respiratory Syndrome Coronavirus (MERS-CoV)

Middle East Respiratory Syndrome Coronavirus (MERS-CoV) emerged in Saudi Arabia in 2012, causing alarming respiratory illnesses. As of September 2020, MERS-CoV has been confirmed as a cause of 2494 illnesses and 858 deaths in 27 countries. It is recognized to be enzootic in camels [7]. This RNA virus belongs to the *Coronaviridae* family.

### 1.8. Enterovirus D68 (EV-D68)

EV-D68 was first isolated in California in 1962, causing infrequent cases in the USA and only minor outbreaks in Europe, Africa, and Southeast Asia. In 2014, however, a novel clade of a 2nd EV-D68 was implicated in a series of outbreaks of respiratory disease across 21 countries, resulting in at least 2529 EV-D68 illnesses and 17 deaths in that year alone. Additional international outbreaks were identified in 2016 and 2018, as the virus was implicated as the cause of severe respiratory illness and acute flaccid myelitis, a polio-like illness. This RNA virus is not thought to be zoonotic. It belongs to the *Picornaviridae* family.

### 1.9. Severe Acute Respiratory Syndrome Coronavirus 2 (SARS-CoV-2)

SARS-CoV-2, a novel bat-like coronavirus, emerged in China in December 2019, and quickly spread worldwide. It is the cause of our current COVID-19 pandemic. Case counts and fatalities continue to increase, having reached 120 M and 2.6 M, respectively, as of 15 March 2021. The virus is thought have originated in bats and moved to man via pangolins, or possibly another, yet-unidentified, intermediate animal host. This RNA virus belongs to the *Coronaviridae* family.

## 2. Economic Costs of Emerging Infectious Diseases

Emerging and reemerging viruses have had major economic impacts. These costs arise from missed work due to worker morbidity and care of loved ones, lost workers due to mortality, healthcare costs, and economic costs of mitigation, which can include disruptions to travel and trade, closing of public spaces and businesses, and compliance with workplace regulations. The World Bank has estimated that the SARS-CoV epidemic cost the affected nations USD 30–50 billion, and the H1N1 pandemic USD 45–55 billion [25]. An incursion of MERS-CoV from June 2015 to June 2016 was estimated to cost South Korea USD 2.6 billion [26]. At present, the economic impact of COVID-19 promises to exceed the combined cost of all other recent respiratory disease epidemics. 

## 3. Predicting Novel Pathogen Emergence

A number of infectious disease modeling teams have worked to identify risk factors for emerging influenza A viruses [27], emerging infectious diseases [28], and emerging zoonotic diseases [29]. The identified risk factors for specific viruses are often complex and sometimes disparate. However, they typically point to densely populated geographical areas in warm regions as areas of high risk. They also point to encounters with live animals, especially in markets and large domestic animal farms [30,31,32,33], as a source of human infection risk. 

We posit that, especially for respiratory viruses, the highest probability of a novel virus emerging occurs in large dense populations of animals, which can sustain the replication of multiple virus subtypes that can recombine. Over time, these viruses and generate new progeny viruses. We see these populations in two contexts: in domestic meat production animals, where a continued introduction of immunologically naïve animals enables multiple viral types to circulate indefinitely in the production animal population, and among mammals that live in large colonies, such as many species of bats. Although their contact with humans is less frequent than the contact domestic animals have with humans, bats often connect with domestic animals, which can serve as intermediate animal hosts in introducing novel viruses to humans. Multiple species of bats have unique immune systems capable of harboring viruses for long periods of time without causing significant illness to their bat colonies, yet they are quite capable of transmitting these same viruses to other animal species. They often do so via roosting in large colonies in habitats such as caves, where enzootic viruses are shared within their population, and by having a long lifespan, showing high geographical movement through flight, and exposing other animal species through fecal and urinary droppings [34].

For some zoonotic wildlife viruses, another factor leading to spillover involves land use change. One recent study, for example, found that land converted to human use had a higher abundance of mammals and birds that serve as reservoirs for zoonotic diseases [35]. Land use change may also influence the emergence of bat-borne emerging diseases through changes to bat geographic ranges, population densities, mixing of different bat species, and their use of human-dominated landscapes for foraging and roosting [36,37]. Thus, human activities can also shape patterns of zoonotic disease risk in nature—including through effects on reservoir host abundance—while also increasing the human–animal contact needed for spillover to occur. More research is needed to understand how these factors influence respiratory virus spillover from wildlife.

## 4. Virus Spillover to Humans Is Common, but Most Viruses Are Poorly Adapted to Humans

A common misunderstanding is that viral spillover to humans is rare, but when it occurs, the virus is fully capable of onward transmission in human populations. Popular novels, movies, and news media promote this view. The reality is different: most animal viruses that humans are exposed to fail to cause significant human infections. The rare virus that does cause a human infection is most often unable to transmit efficiently to other humans. The difficulty of human-to-human transmission occurs because different species often have quite different immunological protections against viral infections, along with different viral receptors on their cells. The new virus may be well-adapted to its current host species but is unlikely to have the unique constellation of viral characteristics that enable it to infect and transmit efficiently in a new host species. 

The phylogenetic proximity of the reservoir host and the new host affects the probability of host shifts [38,39]. This occurs because more closely related hosts will have more similar viral receptors and immunological profiles, along with shared ecological characteristics that facilitate frequent contact. Thus, humans share more parasites and pathogens with the chimpanzee and gorilla than with other primates [40]. Similarly, humans may be more likely to acquire pathogens from captive primates than from other mammals. This effect can also work in reverse, with wild great apes especially susceptible to respiratory viruses from humans [41,42].

Given the many challenges in efficiently transmitting a virus to a new host, significant evolutionary change is typically required for a new virus to adapt to humans and spread from human to human. Viral changes occur via mutations, recombination, or reassortment. Viral mutations are so regular that evolutionary biologists can compare viral sequences and accurately predict how long ago two viruses shared a common ancestor. However, most changes to the viral genome do not increase viral fitness in each host; most mutations result in decreased fitness of the virus, and thus are lost through natural selection. In addition, those mutations that would increase fitness in another host are not under selection pressure until after spillover occurs, making pre-adaptation to other hosts highly unlikely in the original “source” host.

**Figure 1:**
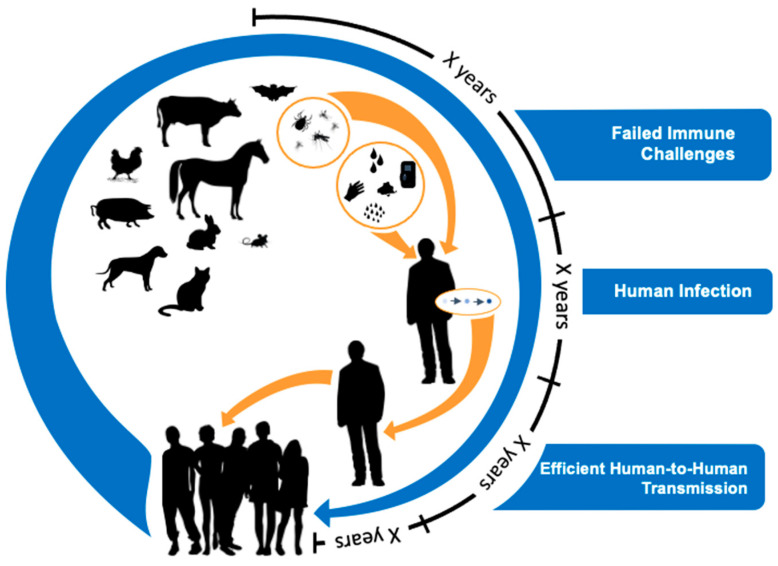
A model for zoonotic pathogen genesis. Viral evolutionary theory suggests the generation of novel animal viruses often fail to cause infection in humans. It is a rarity that a virus will succeed in colonizing or infecting a human. For that virus or its progeny to cause limited human-to-human infection will be rarer still. Finally, it will be incredibly rare for that virus or its progeny to move from limited human-to-human transmission to become highly transmissible among humans. The selection of such animal viruses that become human pathogens is likely to occur incrementally over long periods of time. Hence, evolutionary theory supports the notion that “time is on our side” if we conduct surveillance for novel respiratory viruses at the animal–human interface where this viral “pinging” is occurring. This would be an effective strategy to detect prepandemic viruses before they fully adapt to humans and become highly transmissible. By repeatedly surveilling both animals and humans, one can determine which viruses are becoming successful in adapting to humans and subsequently develop interventions to mitigate transmission.

For a novel virus to adapt to humans generally means that the virus must both successfully continue to replicate in the animal host while also having an affinity for cellular attachment, invasion, and replication in the new human host. Thus, it takes time and the generation of many novel viruses before a virus emerges that is effective in transmitting from human to human. We illustrate this spillover process with a graphic (Figure 1) showing the lengthy time periods required for this incremental process. 

## 5. Evolutionary Perspectives on Efficient Human-to-Human Transmission

These considerations suggest that the evolution of efficient human-to-human transmission requires repeated exposure, involving many people, animals, and settings. Probability-wise, many viruses must first spill over to humans and yet not cause human-to-human transmission before a virus is selected with the fitness to cause limited human-to-human transmission. For a novel virus to become highly transmissible between humans is an extremely rare event (Figure 1). Again, numerous viruses with limited human-to-human transmission are likely to fail before a novel virus has the fitness to cause efficient human-to-human transmission. 

The basic reproductive number (R_0_) is an ecological concept that applies to the reproduction of any organism; it measures the success of a population as the rate of births divided by the rate of deaths. In the context of infectious diseases, this concept translates to the rate at which new infections arise (as a function of contact rates, population density and transmissibility) divided by the rate at which infections are lost (due to disease associated mortality, or virulence, and recovery from infection). R_0_ must be greater than 1 for a virus to invade a fully susceptible novel host population, such as humans, but the challenges just noted mean that most viruses have an R_0_ less than 1 when initially introduced. 

Modeling research has shown that when evolution is considered, even pathogens with an R_0_ that is initially less than 1 can invade a population [43]. This research simulated the transient infections we have been discussing: the occasional spillover of a virus that is not yet adapted to its new host (and thus has an R_0_ < 1). The authors assumed that mutations can occur with some probability that would boost the R_0_ to be greater than 1. The simulations revealed that stuttering chains of transmission occur even when R_0_ < 1, and these chains are longer (resulting in more infected individuals) as R_0_ approaches 1. Moreover, a longer chain of transmissions increased the opportunities for the mutation to occur. In other words, the probability of an adaptive mutation occurring is higher as R_0_ approaches 1. The main message is that anything that facilitates these initial transmission events is also facilitating the evolution of more transmissible variants. 

This consideration of initial transmission chains and evolution also highlights the importance of other factors that can favor the evolution of an efficient virus in humans. The larger the population of the viruses in the new host—in terms of the diversity of viruses, their prevalence in the host population, and the viral load of each host—the more opportunities for mutations to arise that will confer more efficient transmission. Similarly, any factors that increase the length of a transmission chain—such as early super-spreading events or initial infections in chains of immunosuppressed hosts [44]—will also increase opportunities for the necessary mutations to occur, which then increases the probability of adaptive evolution and a large outbreak (or even a pandemic).

While novel animal viruses are assaulting the immune systems of humans with animal exposure, the humans’ immune systems are not static. The immune system, too, is adapting to exposures and increasing immunological resistance as viral insults increase. We see this clearly in seroepidemiological studies of animal-exposed workers with respect to zoonotic influenza A exposures [45]. Animal workers often have elevated antibodies against multiple strains of influenza A virus. Even so, sometimes the magnitude of the viral exposure (viral load) overwhelms the animal workers’ immune systems, and we see animal workers infected despite having preexisting antibodies against enzootic animal viruses [46]. 

This competition between changes in animal viruses and changes in animal workers’ immune defenses is known as antagonistic coevolution [47,48]. This process refers to the reciprocal changes in host and pathogen traits as the two lineages adapt to one another under negative-frequency-dependent selection. Each defensive action by the host can be countered by new mutations in the pathogen to overcome that defense, with selection thus favoring hosts that have new (rare) defenses and favoring pathogens that can infect hosts with the most common defensive strategy. This results in cycles of coevolution, with the host and pathogen continuously evolving. Given the vastly shorter life cycle of viruses, they often have the upper hand in this contest when examined genetically. However, the rapid response of the human immune system enables the animal worker to safely withstand the new viral threats. 

Another evolutionary question involves virulence, which is defined by evolutionary biologists as the damage caused to the host by infection with a pathogen. Traditionally, evolutionary biologists proposed that a highly virulent pathogen is a poorly adapted one. More recent work has shown that this is not the case, and that a variety of factors favor higher virulence [49,50,51]. Virulence is now commonly understood as tradeoff between transmissibility and the death or recovery of a host, which is easily seen in the equation for R_0_ described above. Thus, mutations that influence transmission more than the disease-associated death rate will increase R_0_ and be favored by natural selection. This might be achieved, for example, by mutations that increase viral load, which would be expected to increase transmissibility and host death rate (through use of host resources).

## 6. How Do We Currently Search for Novel Prepandemic Viruses? 

In the United States, public health efforts at mitigating emerging infectious disease threats often vacillate with public interest [52]. Money will be dedicated to investigating and controlling emerging novel virus problems, but, over time, the interest and support eventually wanes [52]. We need to find better ways to establish and sustain worldwide surveillance for novel emerging respiratory virus threats. We need innovative and cost-efficient approaches that will be embraced by both the developed and the developing world. We need to conduct rapid risk assessments when novel viruses are detected and to mitigate their threat to humans and animals once discovered. 

If we conduct surveillance for animal viruses among animal workers, we are likely to be more efficient in identifying pre-pandemic viruses, in contrast to attempting to study all viruses detected in many animal species. Detecting an animal virus in the upper airway of an animal worker already demonstrates some risk to man and makes focusing on further studies of that virus worthwhile. 

**Figure 2:**
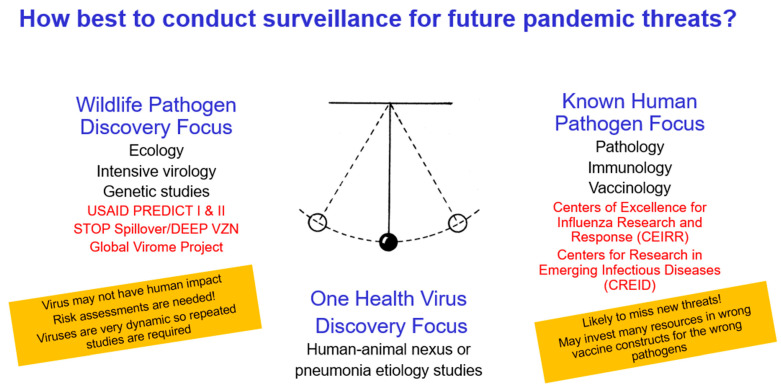
A graphic depicting three strategies for detecting prepandemic pathogens. The *Wildlife Pathogen Discovery Focus* has been embraced by USAID with its PREDICT I and II, STOP Spillover, and DEEP VZN projects, as well as a very ambitious proposed Global Virome Project. It has been criticized as not having precise human illness relevance. The *Know Human Pathogen Focus* has been embraced by the National Institute of Allergy and Infectious Diseases (NIAID) and the Centers of Disease Control and prevention (CDC) in their various centers and large pathogen-centric research teams. It, too, missed foreseeing the 2009 and 2020 pandemic threats. While we recognize that both of these two rather polar approaches have greatly helped in the response to respiratory virus epidemics and pandemics, we argue that a more efficient, cost-effective, and sustainable approach is conducting surveillance for novel respiratory virus threats in geographical hotspots using a One Health approach. Should such studies not be possible, alternatively, pneumonia etiology studies should be performed in these same geographical hotspots to identify emerging respiratory viruses.

In contrast, much of the U.S. effort towards prepandemic virus detection has had a *Wildlife Pathogen Discovery Focus* (Figure 2). Examples include USAID’s PREDICT I and II programs, as well as the proposed Global Virome Project. These programs have been criticized as poorly targeted, expensive and not likely to yield human health benefit (Figure 2). Their costs are also not considered to be sustainable. More than USD 200 M has already been invested in the PREDICT programs since 2009. The proposed Global Virome Project has a projected cost of USD 3.4B, which is considered prohibitive [53,54,55,56,57]. USAID’s new multimillion dollar effort (referred to as “STOP Spillover”) and newly proposed DEEP VZN [58] surveillance and training strategies seem to again be largely focused upon the *Wildlife Pathogen Discovery Focus*.

An alternative to the *Wildlife Pathogen Discovery Focus* strategy has been the *Known Human Pathogen Focus* (Figure 2), which has been embraced by leaders at the US National Institute of Allergy and Infectious Diseases (NIAID) and Centers for Disease Control and Prevention (CDC) for multiple years. NIAID has supported, and continues to support, multiple groups of researchers focused on specific pathogens in their Centers of Excellence for Influenza Research and Surveillance (CEIRS), newly promoted Centers for Research in Emerging Infectious Diseases (CREID), and the expected Centers of Excellence for Influenza Research and Response (CEIRR). NIAID leadership has also proposed large 20-year human-cohort studies, with intensive immunology and pathology research involving 120 known human pathogen groups [59,60,61], although senior leadership has admitted that the large amount of funding necessary to pursue this strategy is not available [59]. The CDC has also long embraced a *Known Human Pathogen Focus* in the organization of its public health and research professionals. Some of these teams are quite large and have a worldwide footprint. A good example is CDC’s Influenza Division, which has more than 300 professionals working across more than 200 surveillance sites, many in international settings.

While there have been benefits to the US government’s efforts in both its *Wildlife Pathogen Discovery Focused* and *Known Human Pathogen Focused* surveillance and research efforts, one can argue that these efforts were not effective in *anticipating* either the 2009 influenza A H1N1 pandemic or the 2020 SARS-CoV-2 pandemic. Fortunately, these efforts were very useful in *responding* to these pandemics. In fact, both strategies have contributed mightily to our successful responses. However, responding well is not ideal, as each pandemic has had tremendous morbidity, mortality, and economic costs. We posit that a more strategic approach to detecting and mitigating prepandemic respiratory viruses would be to concentrate more targeted surveillance at the human–animal interface, where large populations of animals come into contact with humans, and, in doing so, employ a *One Health Approach* [62] in this more targeted surveillance effort (Figure 2). 

The *One Health Approach* involves professionals from disparate disciplines working together to solve complex health problems, such as zoonotic disease epidemics, antimicrobial resistance, and food safety. The goal is to achieve optimum health for humans, animals, and the environment. The *One Health Approach* has been endorsed by numerous academic [63,64,65,66,67,68] and professional organizations [69,70,71,72,73], governments, and health-concerned multilateral organizations, including the WHO, FAO, OIE, the World Bank and the G20.

We recognize that USAID, NIAID, the CDC, and most other US governmental agencies publicly embrace the *One Health Approach* and invest some effort in One Health activities. However, we argue that, when compared to their *Wildlife Pathogen Discovery Focused* or *Known Human Pathogen Focused* surveillance and research efforts, their investment in the *One Health Approach* is quite modest. By periodically examining both animals and humans using a *One Health Approach,* we would be able to determine which viruses are becoming successful in adapting to humans and subsequently develop interventions to stop that viral adaptation. This could save millions of lives and prevent tens of millions of infections regarding a prepandemic virus threat. 

Viral evolutionary theory suggests the majority of novel animal viruses often fail to cause infection in humans. It is a rarity that a virus will succeed in colonizing or infecting humans. For that virus or its progeny to cause limited human-to-human infection will be rarer still. It will be incredibly rare for that virus or its progeny to become highly transmissible among humans. The selection of such viruses is likely to occur incrementally over long periods of time. Hence, evolutionary theory supports the notion that conducting surveillance for novel viruses at the animal–human interface would be an effective strategy to detect prepandemic viruses before they fully adapt to humans and become highly transmissible.

Alternatively, if One Health surveillance for prepandemic respiratory viruses is not possible at the human–animal interface, a second high-yield strategy would be to conduct surveillance for novel animal pathogens among pneumonia patients in geographical areas known to be at high risk of novel respiratory virus emergence. We have recently applied these strategies with good success and at modest cost in detecting zoonotic coronaviruses among humans hospitalized with pneumonia (Malaysia) [74], and recently isolated and fully sequenced a recombinant, canine-like, feline-like, alphacoronavirus from among these patients [personal communication G. Gray]. We have also detected a bat-like adenovirus in a patient with respiratory illness (Malaysia) [75]. Other teams have similarly found evidence of animal coronaviruses in humans (USA, Haiti) [76,77].

Using the *One Health Approach,* we have documented human metapneumovirus infections in turkeys and evidence for avian pneumovirus infections among turkey workers (USA) [15,16]. We have also found molecular evidence of influenza D virus among poultry (Malaysia) [78], human enteroviruses in pig slurry (USA) [79], and equine influenza A virus spillover from horses to camels (Mongolia) [80].

Successful international human–animal interface network studies will necessarily often require the identification of primary partners in emerging infectious disease hotspots among national or regional veterinary institutions. Such institutions often have better access to environments where animal workers are likely to be exposed to enzootic animal respiratory viruses. For pneumonia network studies, this will involve referral hospital institutions with a relatively high volume of pneumonias. Fundamental to both human–animal interface and pneumonia networks surveillance is the identification of laboratories with the molecular equipment and ultracold freezers necessary to detect and preserve respiratory viruses. 

## 7. What Would Be the Best Strategy to Detect Novel Prepandemic Viruses 

Having argued that we should conduct One Health surveillance at the human–animal interface, specifically where and how should we do so? Some have argued that we should focus surveillance on novel respiratory viruses among humans who have contact with wildlife, especially bush meat hunters and wet market workers in geographical areas rich in biodiversity. However, hominids have been eating bushmeat for several hundred thousand years, and only recently have we begun to note zoonotic respiratory virus epidemics. One could argue that while such bushmeat hunters or marketers may occasionally be infected with a wildlife virus, the virus or its progeny are not likely to have the prolonged exposure to humans required to become highly transmissible to man. 

A more logical approach is supported by evolutionary and ecological data. For instance, in their 2009 report, Lloyd-Smith and colleagues [81] present their concept of “spillover force of infection”, which they describe as the probability of spillover as dependent on three major components: (1) prevalence of the pathogen in the reservoir, (2) the reservoir-to-human contact rate, and (3) the probability of human infection with the animal pathogen. If one considers areas where these components are high, one may effectively select the best sites for conducting surveillance of pre-pandemic viruses.

### 7.1. Among Which Animals Is Prevalence of Respiratory Viruses High?

If we consider the five viral families of respiratory viruses discussed earlier in this text, data are available to answer this question for, at least, influenza A viruses (*Orthomyxoviridae*) and coronaviruses (*Coronaviridae*). The published literature is not rich with prevalence data among animals for the other viruses of concern. Influenza A viruses are highly enzootic in livestock, especially poultry and swine, as well as among aquatic birds. Influenza D viruses are also thought to be highly enzootic in cattle and swine [82]. Coronaviruses are enzootic in numerous animal species, especially in bats, birds, and livestock [83]. Hence, studying these animal species seems an appropriate strategy. Additionally, studying dense, dynamic populations of these animals with efficient reproduction is very strategic, as such animal populations will be able to sustain respiratory virus populations via continual infections among immunologically naïve offspring.

### 7.2. Where Is the Animal Reservoir-Human Contact Rate High?

If we consider the bats and domestic animals mentioned above, we might select sites for surveillance where these animals have frequent and close contact with man. Bats are not frequently in direct contact with humans, save for those occasional colonies which are maintained in zoos, for research purposes, or by bat enthusiasts. In contrast, domestic livestock, such as pigs, chickens, ducks, geese, cattle, goats, and sheep, are often reared in close contact with humans. Modern industrial farming efforts are increasing the size of such domestic herds or flocks, as their meat can be produced more efficiently and at lower cost compared to farms with smaller herds or flocks. The downside of these large industrialized farms is their propensity to harbor sustained animal pathogens among livestock, which may cause mild disease in the animals but, over time, can cross-over to infect animal workers. Reports of novel pathogen generation on industrialized farms and transmission to humans is becoming increasingly common for both viral and bacterial pathogens [84,85,86,87]. Consistent with the logic prescribed by Lloyd-Smith and colleagues [81], large industrialized farms in emerging infectious disease hotspots would be strategic sites for conducting surveillance for novel prepandemic respiratory viruses. 

### 7.3. From Which Viruses and Viral Hosts Is the Probability of Zoonotic Virus Transmission to Humans Highest?

Again considering the five viral families of concern, data to support an answer to this question are a bit sparse. However, some data are available. Freidl et al. [88] published a large review of animal to human risk for zoonotic influenza A in 2014. After reviewing 89 published reports, they concluded that the risk was greatest from human exposure to poultry and pigs. In our 2019 review of the literature for zoonotic influenza A transmission to humans [89], we concluded that the species barrier is lower for viruses moving from swine to humans rather than from other animals to humans. In a 2019 report, Wang et al. [90] concluded that the probability of interspecies transmission of coronaviruses from two different coronavirus genera, alphacoronaviruses, and deltacoronaviruses are especially high. Given that pigs have experienced recent epizoonotics from five novel coronaviruses [83], it seems worthwhile to conduct surveillance for prepandemic coronaviruses among pigs. Our research team has recently conducted reviews of the literature regarding adenovirus [23] and enterovirus [21] zoonotic infections. While data are sparse, it seems relevant that nonhuman primates might be at the greatest risk of sharing adenoviruses and enteroviruses with humans.

## 8. Efficient Field Collection, Clinical and Laboratory Methods

**Figure 3:**
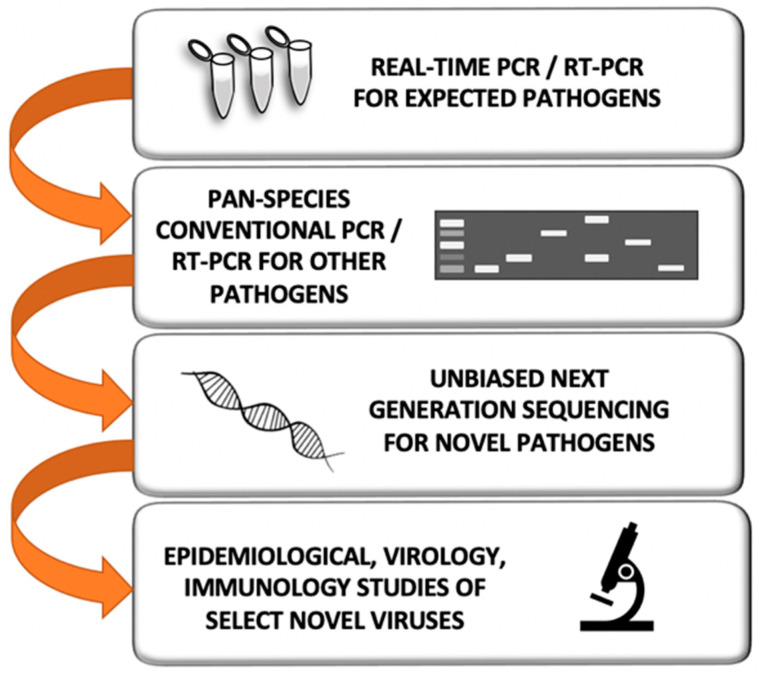
Flow diagram for field specimen study. Our international partners conduct most of the real-time RT-PCR/PCR screening work and some of the pan-species molecular work. If a virus is not yet identified but suspected, next-generation sequencing may be employed for pathogen discovery. Where we see evidence of the same respiratory viruses in both the animal workers and their animals, we next intensely study those viruses for their prepandemic potential. This may involve attempts at viral culture in multiple human and animal cells lines and full genome sequencing. If further risk assessment is warranted, this may involve seroepidemiological studies of other animal workers, the development of specific molecular assays for geographical prevalence studies, and pathogenesis studies in animal models.

Ideally, laboratory work should be designed such that partners in conducting human-animal nexus or pneumonia etiology surveillance networks are trained with standard operating procedures (SOPs) that are adapted to their field, hospital, and laboratory environments. This will often require the shipment of quality reagents and conducting capacity-building workshops. International partners should be trained to conduct as much of the field sampling, clinical sampling, and laboratory assays as they can. As the collaboration progresses, partners may wish to become more and more autonomous. We have had considerable success in carrying out such partnered surveillance work. In general, we anticipate our field sites to collect, process, and minimally study specimens with real-time molecular assays (Figure 3), after which specimens are shipped to one of our three research laboratories (China, Singapore, or the United States), where additional studies are performed as described below.

We approach our international partners with options regarding what type of specimens they wish to collect. For human–animal interface studies, this often involves nasal washes or nasopharyngeal swab, and sometimes fecal specimen collections from the workers’ animals. We often also collect serial serum specimens from the animal workers. We typically train international partners in collecting environmental samples, including bioaerosol, water, and animal environmental samples.

**Figure 4:**
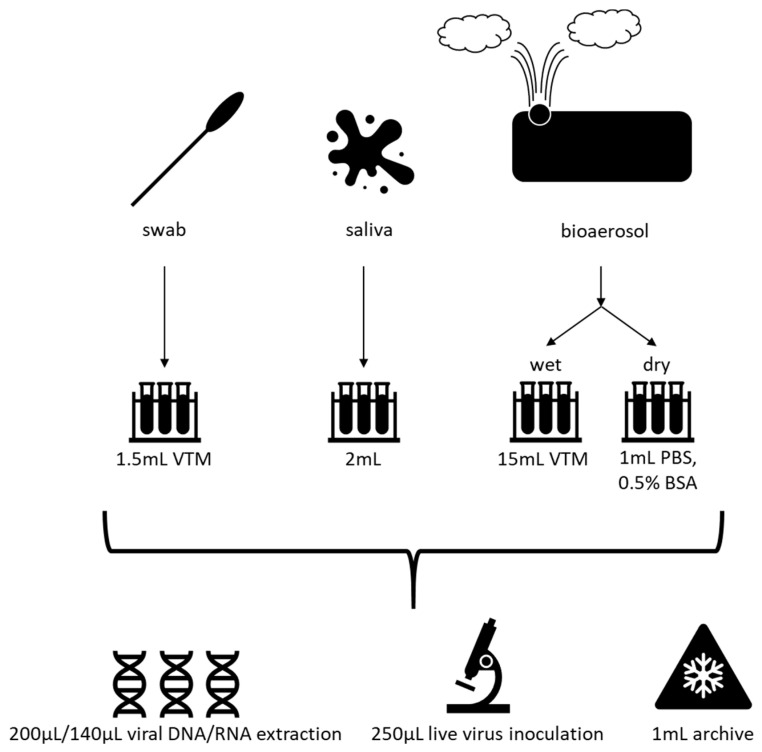
Sample processing workflow. Swab samples are collected in 1.5 mL viral transport media (VTM), 2 mL of saliva is collected as-is, and bioaerosol samples are collected in a wet stage (15 mL VTM) or a dry stage, which is then suspended in 1 mL phosphate-buffered saline (PBS) with 0.5% bovine serum albumin (BSA). Subsequently, these samples are divided for DNA/RNA extraction, inoculation in cell culture to obtain live virus, or stored as an archival sample at −80 °C.

Biological and environmental swabs are typically collected with a commercially available swab kit, though we recommend reducing the viral transfer medium (VTM) by up to half for samples that are suspected to have low viral presence. Following the collection of samples in the field, specimens are brought to the lab for processing, with the protocol dependent on the type of sample collected. Nasopharyngeal, rectal, and fomite swabs are generally collected in 1.5 mL viral transfer medium (VTM) or utilizing a commercially available swab kit (Figure 4). Around 2 mL of saliva is collected as-is. For aerosol sampling, we most often use National Institute of Occupational Safety and Health (NIOSH) two-stage bioaerosol cyclone samplers connected to a SKC AirCheck Tough personal sampling pump (Cat # 220-50000TC-K; SKC Inc. Eighty Four, PA, USA) [91]. Samples are collected in two stages: a liquid stage (15 mL in VTM), and a dry stage, and then suspended in 1 mL PBS containing 0.5% bovine serum albumin. These samples are aliquoted, reserving 140–200 µL for viral DNA/RNA extraction, 250 µL for live virus inoculation (stored at −80 °C), and 1 mL as an archival sample (stored at −80 °C) (Figure 3).

**Figure 5:**
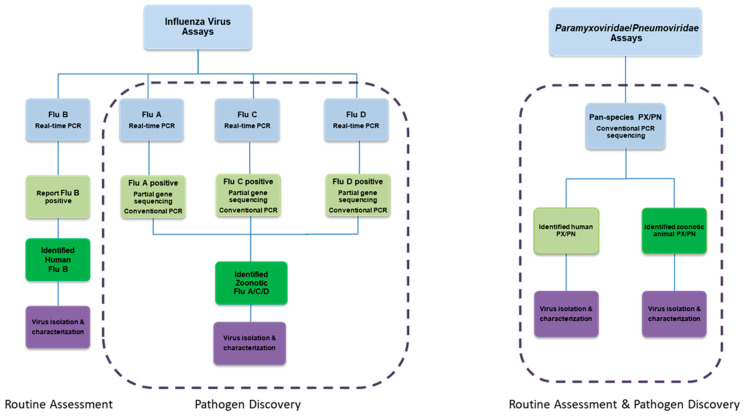
Molecular detection algorithm for influenza viruses and pan-*Paramyxoviridae*/*Pneumoviridae* viruses. The influenza virus assay utilizes real-time (q) RT-PCR to differentiate the various strains of influenza virus. For influenza A, C, and D viruses, this is followed by conventional RT-PCR assays for subtyping. In contrast, the *Paramyxoviridae*/*Pneumoviridae* algorithm begins with a pan-species conventional RT-PCR approach to identify human and animal viruses of the *Paramyxoviridae* and *Pneumoviridae* families. Viruses are further characterized with Sanger sequencing.

Viral nucleic acid extractions are subjected to a variety of molecular-detection algorithms. These assays incorporate routine assessment and pathogen discovery pathways for specific viral families. Initially, these assays use algorithms of real-time PCR (qPCR or qRT-PCR) and conventional PCR/RT-PCR assay techniques to detect previously identified viruses and novel viruses within that same group. For instance, for our influenza A virus group we often search for influenza A, B, C, and D with qRT-PCR assays; when a virus is detected, we employ conventional RT-PCR with Sanger sequencing to further characterize the virus (Figure 5) [92,93,94,95]. For some virus groups, we begin with a pan-species diagnostic approach (such as with our conventional pan-*Paramyxoviridae*/*Pneumoviridae* RT-PCR assay (Figure 5) [96]) and then further characterize individual viruses through Sanger sequencing of the amplicon. This permits us to identify viruses of both human and animal origin (Figure 5).

**Figure 6:**
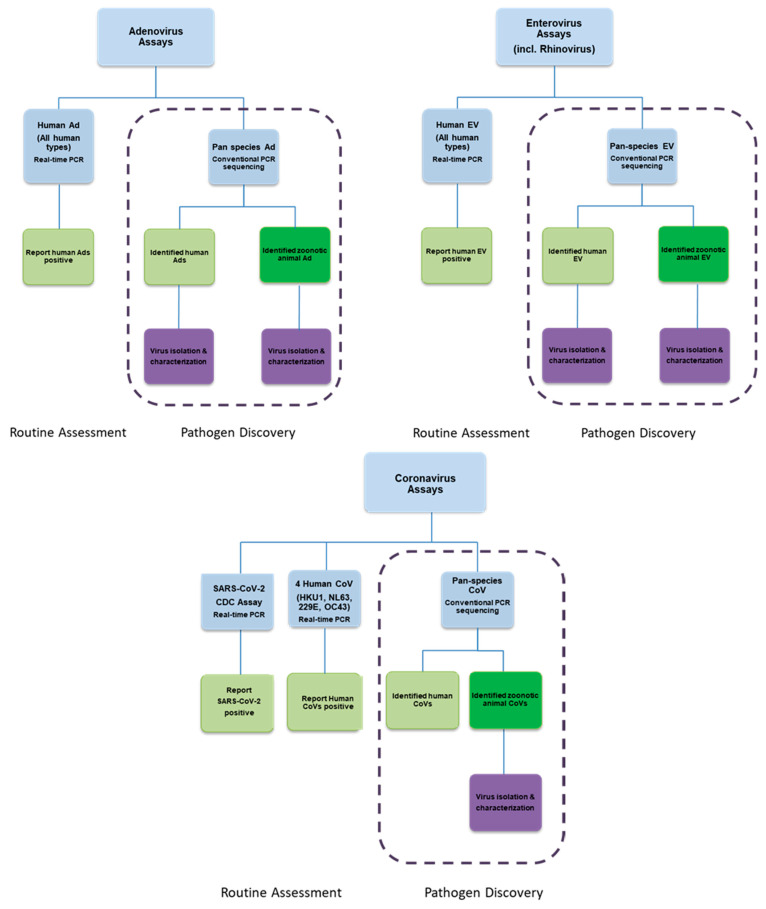
Molecular detection assays for adenovirus (Ad), enterovirus (EV), and coronavirus (CoV). In these algorithms, we combine qPCR/qRT-PCR, and conventional pan-species PCR/qRT-PCR work to detect and characterize both previously recognized and novel human or animal viruses.

Molecular detection algorithms for adenoviruses and enteroviruses incorporate both qPCR/qRT-PCR to identify human strains of these viruses and conventional PCR in a pan-species RT-PCR approach, with sequencing to characterize novel human and animal virus strains (Figure 6) [97,98,99,100,101]. Similarly, for our coronavirus algorithm, we employ a battery of specific qRT-PCR assays to identify previously known seasonal or pandemic human coronaviruses (Figure 6) [102] and a pan-species coronavirus conventional PCR assay to detect and characterize other human or animal coronaviruses [74]. These assays were expanded to incorporate detection of the SARS-CoV-2 virus in biological and environmental samples during the onset of the 2020 pandemic [103]. Detailed versions of each assay can be found in the Supplementary Tables and Figures associated with this manuscript. Overall, these assays are vital in detecting and characterizing both previously recognized respiratory viruses and novel emerging viruses that would likely be missed by commercial assays. 

## 9. Novel Virus Risk Assessments

As part of our pathogen discovery pathway, field specimens with evidence of a novel virus are transported to one of our dedicated research laboratories for further study. This may involve work to determine if a novel virus meets Bradford Hill or other related criteria to be considered as a human pathogen. Often, characterization involves assessing viral infectivity in various cell lines to support hypotheses for host range and pathogenicity. From the original specimen, 250 µL of the sample is inoculated in an appropriate cell line. This procedure requires previous knowledge relating to characteristics of the viral family to determine which cell culture line would provide the optimal conditions for growing the virus. Cytopathic effects (CPE) are monitored in the inoculated cells to assess successful viral amplification, and virus in the supernatant is harvested when 80% CPE is observed. 

We next develop a real-time molecular assay for that pathogen. The resultant virus is confirmed to grow in cell culture using qPCR/qRT-PCR, and the virus concentration is titered. In the case of novel viruses, the DNA/RNA may be extracted and sent for whole-genome sequencing. Isolated viruses are stored for future use by our team or apportioned to other researchers through our online biorepository. If the virus is especially threatening it may be necessary to determine the virus’ geographical and host prevalence. This may involve the development of a serum neutralization assay and seroepidemiological studies of animal workers and their animals. Further concerns may lead to pathogenesis studies in animal models.

## 10. Conclusions

In this manuscript, we endeavored to review the most common respiratory virus threats and detail their morbidity. We discussed current efforts at early emerging pathogen detection and noted their recent failure in providing early warning for the 2009 influenza A virus and 2020 coronavirus pandemics. We also proposed an alternate One Health strategy for novel respiratory virus detection and argued that it is likely to be more efficient and more sustainable than current efforts, especially when integrated with research perspectives from ecology and evolution. Finally, we provided considerable detail regarding laboratory methods in employing this One Health strategy. We proposed establishing networks for novel respiratory virus surveillance in known geographical hotspots for emerging infectious diseases. We proposed targeting sites where humans and animals interface, such as live-animal markets, large industrial farms, or alternatively, when that is not possible, large hospital sites with high rates of pneumonia admissions where the etiology of these patients might be studied for novel respiratory virus pathogens. By employing targeted, minimally invasive surveillance studies, future respiratory virus threats may be more effectively detected mitigated prior to causing tremendous human and economic costs to the affected nations.

## Figures and Tables

**Table 1:** Recent epidemics of emerging or reemerging respiratory viruses.

Years	Virus	Geographical Range	Estimated Morbidity (Infections)	Estimated Mortality (Deaths)
1995+	Human metapneumovirus ^†^ [1]	Worldwide	42,464	At least 41 (Table S1)
1995+	Rhinovirus Group C ^†^ [2]	Worldwide	1632	At least 7 (Table S2)
1998+	Enterovirus A71 [3]	Worldwide	12.8 M *	At least 3747 *
2003–2004	SARS-CoV ^†^ [4]	29 countries	8098	774
2006+	Adenovirus 14 [5]	United States, Ireland, China, Canada	>750	At least 13
2009+	influenza A (H1N1)pdm09 virus ^†^ [6]	Worldwide	100.5 M ^‡^	75,000 ^‡^
2012+	MERS-CoV ^†^ [7]	27 countries	2494 **^§^**	858 **^§^**
2014+	Enterovirus D68 [8,9]	Worldwide	2529 ^¶^	At least 17 ^¶^
2019+	SARS-CoV-2 ^†^ [10]	Worldwide	>120,000,000 as of 03/15/2021	>2,659,000 as of 03/15/2021

* Asian-Pacific Region only, 1998–2018; ^†^ Virus is thought to cross species (zoonotic); ^‡^ for years 2009–2018; **^§^** As of September 2020; ^¶^ 2014–2015 SARS-CoV: Severe Acute Respiratory Syndrome Coronavirus; MERS-COV: Middle East Respiratory Syndrome Coronavirus; SARS-CoV-2: Severe Acute Respiratory Syndrome Coronavirus 2.

## Data Availability

Not applicable.

## Supplementary Materials

The following are available online at https://www.mdpi.com/article/10.3390/v13040637/s1. Table S1: Published studies reporting human metapneumovirus (hMPV) related mortalities. Table S2: Published studies reporting Rhinovirus Group C (HRV-C) related mortalities. Figure S1: Detection of influenza A virus using reverse transcription (RT), real-time PCR (qPCR). Figure S2: Detection of influenza B virus using reverse transcription (RT), real-time PCR (qPCR). Figure S3: Detection of influenza C virus using reverse transcription (RT), real-time PCR (qPCR). Figure S4: Detection of influenza D virus using reverse transcription (RT), real-time PCR (qPCR). Figure S5: Detection of influenza A virus using reverse transcription (RT), conventional PCR (convPCR) against all 8 RNA gene segments. Figure S6: Detection of influenza D virus using reverse transcription (RT), conventional PCR (convPCR). Figure S7: Detection of *Paramyxoviridae*/*Pneumoviridae* viruses using reverse transcription (RT), conventional PCR (convPCR). Figure S8: Detection of human adenoviruses using real-time PCR (qPCR). Figure S9: Detection of animal adenoviruses using conventional PCR (convPCR). Figure S10: Detection of pan-adenoviruses using conventional PCR (convPCR). Figure S11: Detection of enterovirus using reverse transcription (RT), real-time PCR (qPCR). Figure S12: Detection of pan-enteroviruses using reverse transcription (RT), conventional PCR (convPCR). Figure S13: Detection of pan-coronaviruses using reverse transcription (RT), conventional PCR (convPCR). Figure S14: Detection of SARS-CoV-2 using reverse transcription (RT), real-time PCR (qPCR).
