# Supplementary material for: Mitigating Future Respiratory Virus Pandemics: New Threats and Approaches to Consider
Source: Viruses. 2021 Apr 8;13(4):637. doi: 10.3390/v13040637 (PMC8068197; doi:10.3390/v13040637)
Supplement: Supplementary file 1 [file viruses-13-00637-s001.pdf]

March 12, 2021

Supplemental Tables and Figures

Mitigating Future Respiratory Virus Pandemics: New Threats and Approaches to Consider

Gregory C. Gray[1,2,3,4*], Emily R. Robie[1,2], Caleb J. Studstill[1,2], and Charles L. Nunn[2,5]

1 Division of Infectious Diseases, Duke University School of Medicine, Durham, NC, 27710 USA; emily.robie@duke.edu (E.R.R.); calebjstudstill@gmail.com (C.J.S.)
2 Duke Global Health Institute, Duke University, Durham, NC, 27710 USA; clnunn@duke.edu
3 Emerging Infectious Disease Program, Duke-NUS Medical School, Singapore, 169856
4 Global Health Center, Duke Kunshan University, Kunshan, China 215316
5 Evolutionary Anthropology, Duke University, Durham, NC, 27710 USA
* Correspondence: Gregory.gray@duke.edu; Tel.: +1-919-684-1032

Table S1. Published studies reporting human metapneumovirus (hMPV) related mortalities.

Source	Time Frame of Sample Collection	Cases of hMPV identified	Deaths attributed to hMPV	Country
Englund JA, Boeckh M, Kuypers J, et al. Brief communication: Fatal human metapneumovirus infection in stem-cell transplant recipients. *Ann Intern Med*. 2006;144(5):344-349.	1995 – 1999	5	4	--
Pelletier G, Déry P, Abed Y, Boivin G. Respiratory tract reinfections by the new human Metapneumovirus in an immunocompromised child. *Emerg Infect Dis*. 2002;8(9):976-978. doi:10.3201/eid0809.020238	1999	1	1	Canada
Falsey AR, Erdman D, Anderson LJ, Walsh EE. Human metapneumovirus infections in young and elderly adults. *J Infect Dis*. 2003;187(5):785-790. doi:10.1086/367901	1999 – 2001	44	1	US
Walsh EE, Peterson DR, Falsey AR. Human Metapneumovirus Infections in Adults: Another Piece of the Puzzle. *Arch Intern Med*. 2008;168(22):2489–2496. doi:10.1001/archinte.168.22.2489	1999 - 2003	241	6	US
Williams JV, Martino R, Rabella N, et al. A prospective study comparing human metapneumovirus with other respiratory viruses in adults with hematologic malignancies and respiratory tract infections. *J Infect Dis*. 2005;192(6):1061-1065. doi:10.1086/432732	1999 – 2004	22	3	Spain
Cane PA, van den Hoogen BG, Chakrabarti S, Fegan CD, Osterhaus AD. Human metapneumovirus in a haematopoietic stem cell transplant recipient with fatal lower respiratory tract disease. *Bone Marrow Transplant*. 2003;31(4):309-310. doi:10.1038/sj.bmt.1703849	2000	1	1	--
Morrow BM, Hatherill M, Smuts HE, Yeats J, Pitcher R, Argent AC. Clinical course of hospitalised children infected with human metapneumovirus and respiratory syncytial virus. *J Paediatr Child Health*. 2006;42(4):174-178. doi:10.1111/j.1440-1754.2006.00825.x	2001 – 2003	17	3	South Africa
Noyola DE, Alpuche-Solís AG, Herrera-Díaz A, Soria-Guerra RE, Sánchez-Alvarado J, López-Revilla R. Human metapneumovirus infections in Mexico: epidemiological and clinical characteristics. *J Med Microbiol*. 2005;54(Pt 10):969-974. doi:10.1099/jmm.0.46052-0	2002 – 2004	34	1	Mexico
Larcher C, Geltner C, Fischer H, Nachbaur D, Müller LC, Huemer HP. Human metapneumovirus infection in lung transplant recipients: clinical presentation and epidemiology. *J Heart Lung Transplant*. 2005;24(11):1891-1901. doi:10.1016/j.healun.2005.02.014	2003 – 2004	43	3	Austria

Sivaprakasam V, Collins TC, Aitken C, Carman WF. Life-threatening human metapneumovirus infections in West of Scotland. *J Clin Virol*. 2007;39(3):234-237. doi:10.1016/j.jcv.2007.03.011	2004 – 2006	206	2	Scotland
Boivin G, De Serres G, Hamelin ME, et al. An outbreak of severe respiratory tract infection due to human metapneumovirus in a long-term care facility. *Clin Infect Dis*. 2007;44(9):1152-1158. doi:10.1086/513204	2006	6	3	Canada
Yong Kwan Lim, Oh Joo Kweon, Hye Ryoun Kim, Tae-Hyoung Kim, Mi-Kyung Lee, Clinical Features, Epidemiology, and Climatic Impact of Genotype-specific Human Metapneumovirus Infections: Long-term Surveillance of Hospitalized Patients in South Korea, Clinical Infectious Diseases, Volume 70, Issue 12, 15 June 2020, Pages 2683 - 2694, https://doi.org/10.1093/cid/ciz697	2007 - 2016	1,275	7	South Korea
Shahda S, Carlos WG, Kiel PJ, Khan BA, Hage CA. The human metapneumovirus: a case series and review of the literature. *Transpl Infect Dis*. 2011;13(3):324-328. doi:10.1111/j.1399-3062.2010.00575.x	2008 – 2009	9	2	US
Holzemer NF, Hasvold JJ, Pohl KJ, Ashbrook MJ, Meert KL, Quasney MW. Human Metapneumovirus Infection in Hospitalized Children. *Respir Care*. 2020;65(5):650-657. doi:10.4187/respcare.07156	2009 – 2013	131	1	US
Liao RS, Appelgate DM, Pelz RK. An outbreak of severe respiratory tract infection due to human metapneumovirus in a long-term care facility for the elderly in Oregon. *J Clin Virol*. 2012;53(2):171-173. doi:10.1016/j.jcv.2011.10.010	2011	6	2	US
Shih, Hsin-I MD, MPH; Wang, Hsuan-Chen MS; Su, Ih-Jen MD, PhD; Hsu, Hsiang-Chin MD; Wang, Jen-Ren PhD; Sun, Hsiao Fang Sunny PhD; Chou, Chien-Hsuan MD; Ko, Wen-Chien MD; Hsieh, Ming-I BS; Wu, Chi-Jung MD, PhD Viral Respiratory Tract Infections in Adult Patients Attending Outpatient and Emergency Departments, Taiwan, 2012–2013, Medicine: September 2015 - Volume 94 - Issue 38 - p e1545, doi: 10.1097/MD.0000000000001545	2012 – 2013	10	1	Taiwan

Table S2. Published studies reporting Rhinovirus Group C (HRV-C) related mortalities.

Source	Time Frame of Sample Collection	Cases of HRV-C identified	Deaths attributed to HRV-C	Country
Miller EK, Edwards KM, Weinberg GA, et al. A novel group of rhinoviruses is associated with asthma hospitalizations. *J Allergy Clin Immunol*. 2009;123(1):98-104.e1. doi:10.1016/j.jaci.2008.10.007	2001 – 2003	77	1	USA
Miller EK, Khuri-Bulos N, Williams JV, et al. Human rhinovirus C associated with wheezing in hospitalised children in the Middle East. *J Clin Virol*. 2009;46(1):85-89. doi:10.1016/j.jcv.2009.06.007	2007	62	1	Jordan
Fuji N, Suzuki A, Lupisan S, et al. Detection of human rhinovirus C viral genome in blood among children with severe respiratory infections in the Philippines. *PLoS One*. 2011;6(11):e27247. doi:10.1371/journal.pone.0027247	2008 – 2009	83	5	Philippines

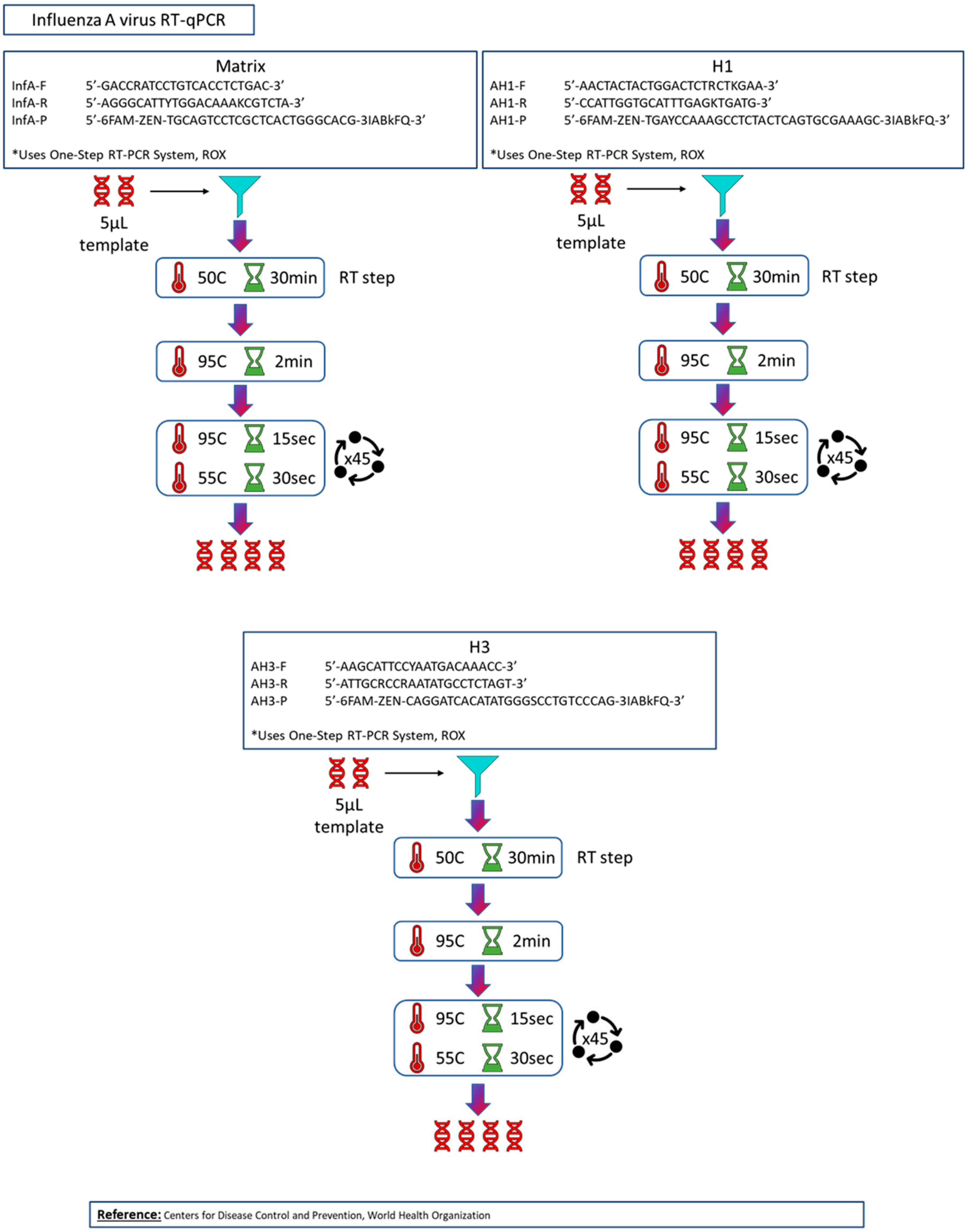

Figure S1. Detection of influenza A virus using reverse transcription (RT), real-time PCR (qPCR). The primers and probe specific to the matrix gene and primers and probes used for the detection of H1 and H3 (subtyping specific influenza A viruses) are shown. This protocol utilized the SuperScript III Platinum One-Step qRT-PCR Kit (Invitrogen). In this assay, 0.4µL forward/reverse primers (40µM), 0.4µL probe (10µM), 0.4µL ROX (1:10 dilution), and 5µL extracted RNA are added to the qPCR reagents. The total reaction volume is 20µL.

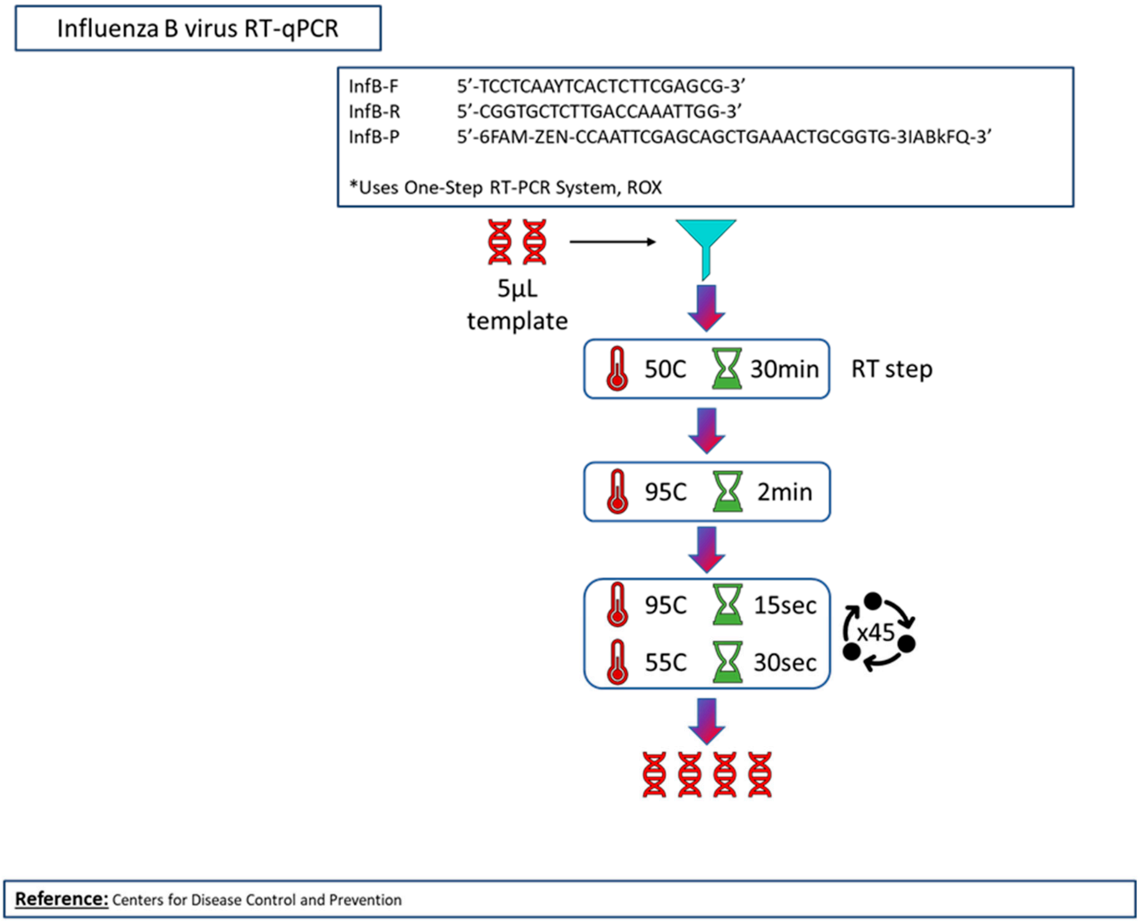

Figure S2. Detection of influenza B virus using reverse transcription (RT), real-time PCR (qPCR). Primers and probe specific to influenza B virus are listed. This protocol utilizes the SuperScript III Platinum One-Step qRT-PCR Kit (Invitrogen). In this assay, 0.4μL forward/reverse primers (40μM), 0.4μL probe (10μM), 0.4μL ROX (1:10 dilution), and 5μL extracted RNA are added to the qPCR reagents. The total reaction volume is 20μL.

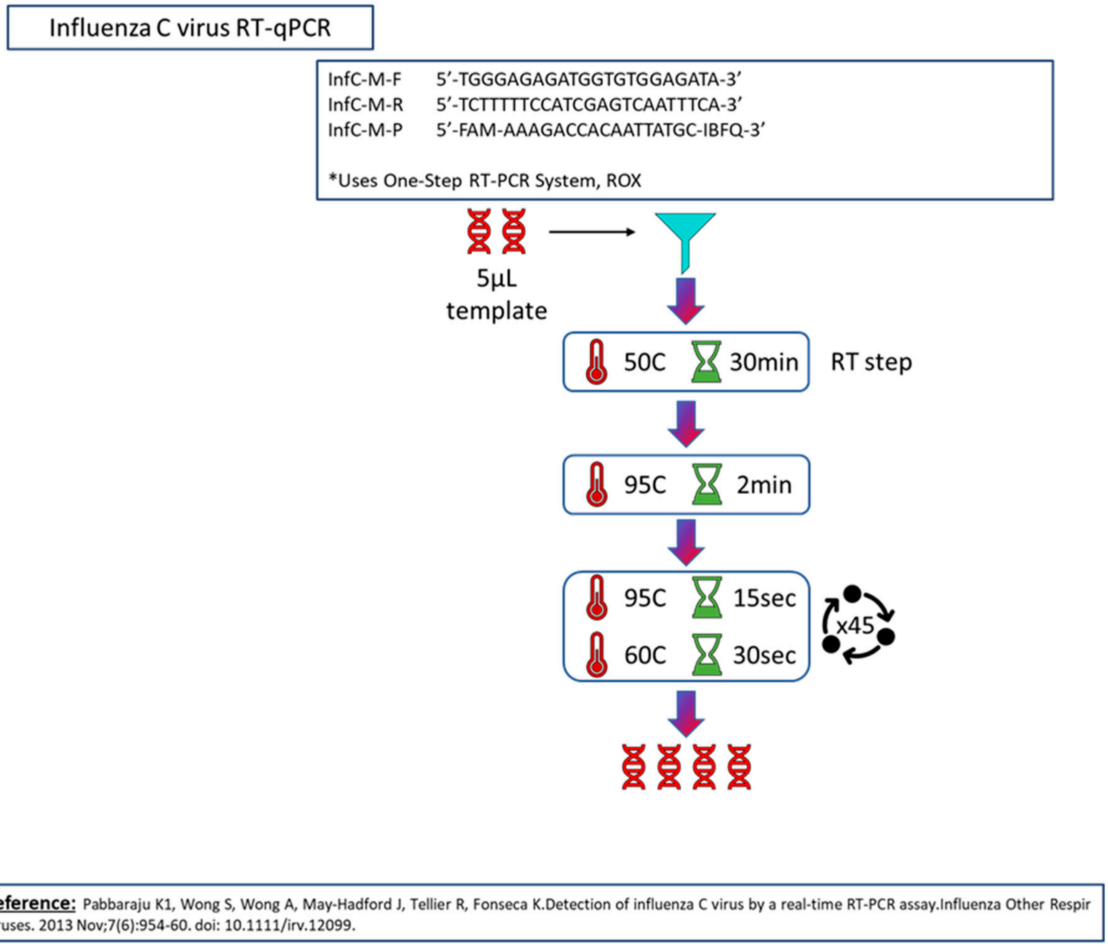

Figure S3. Detection of influenza C virus using reverse transcription (RT), real-time PCR (qPCR) [1]. Primers and probe specific to influenza C virus matrix protein are listed. This protocol utilizes the SuperScript III Platinum One-Step qRT-PCR Kit (Invitrogen). In this assay, 0.4µL primers (40µM), 0.4µL probe (10µM), 0.4µL ROX (1:10 dilution), and 5µL extracted RNA are added to the qPCR reagents. The total reaction volume is 20µL.

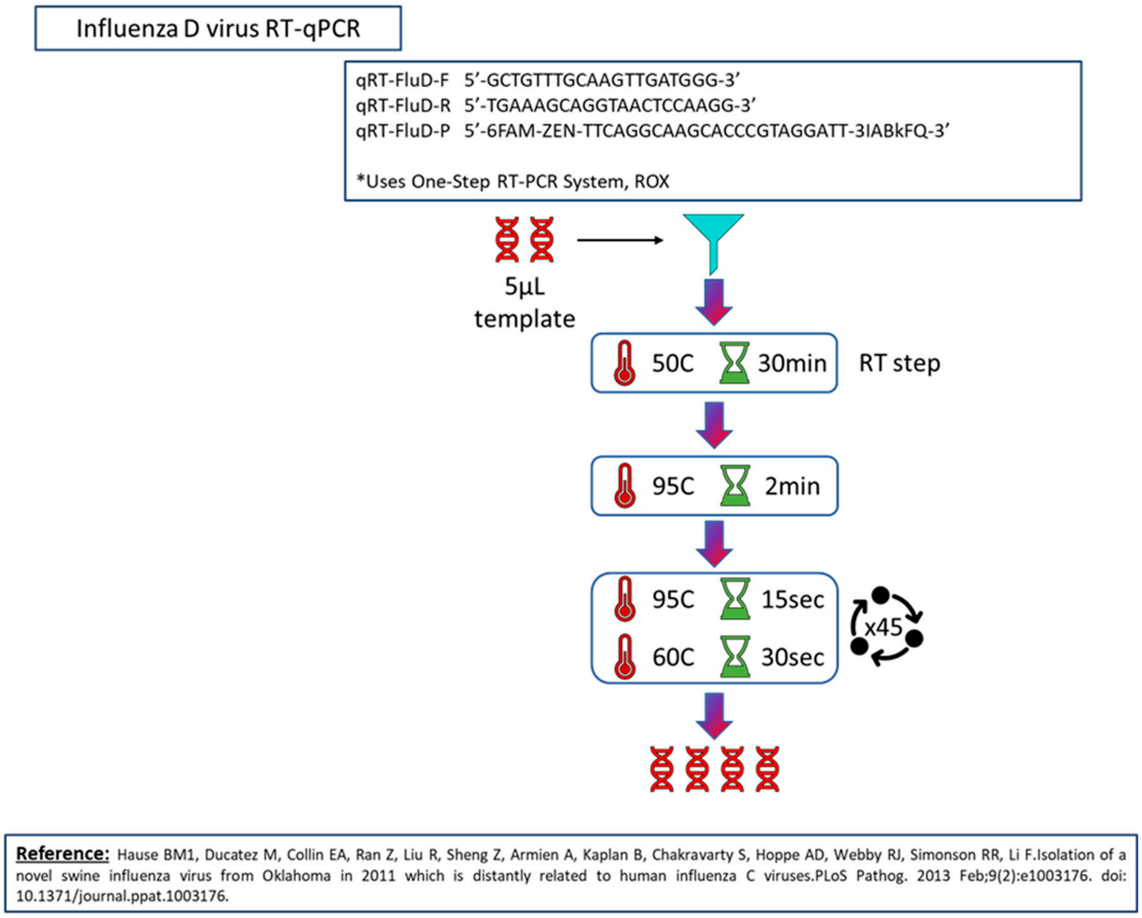

Figure S4. Detection of influenza D virus using reverse transcription (RT), real-time PCR (qPCR) [2]. Primers and probe specific to influenza D virus PB1 are listed. This protocol utilizes the SuperScript III Platinum One-Step qRT-PCR Kit (Invitrogen). In this assay, 0.4μL primers (40μM), 0.4μL probe (10μM), 0.4μL ROX (1:10 dilution), and 5μL extracted RNA are added to the qPCR reagents. The total reaction volume is 20μL.

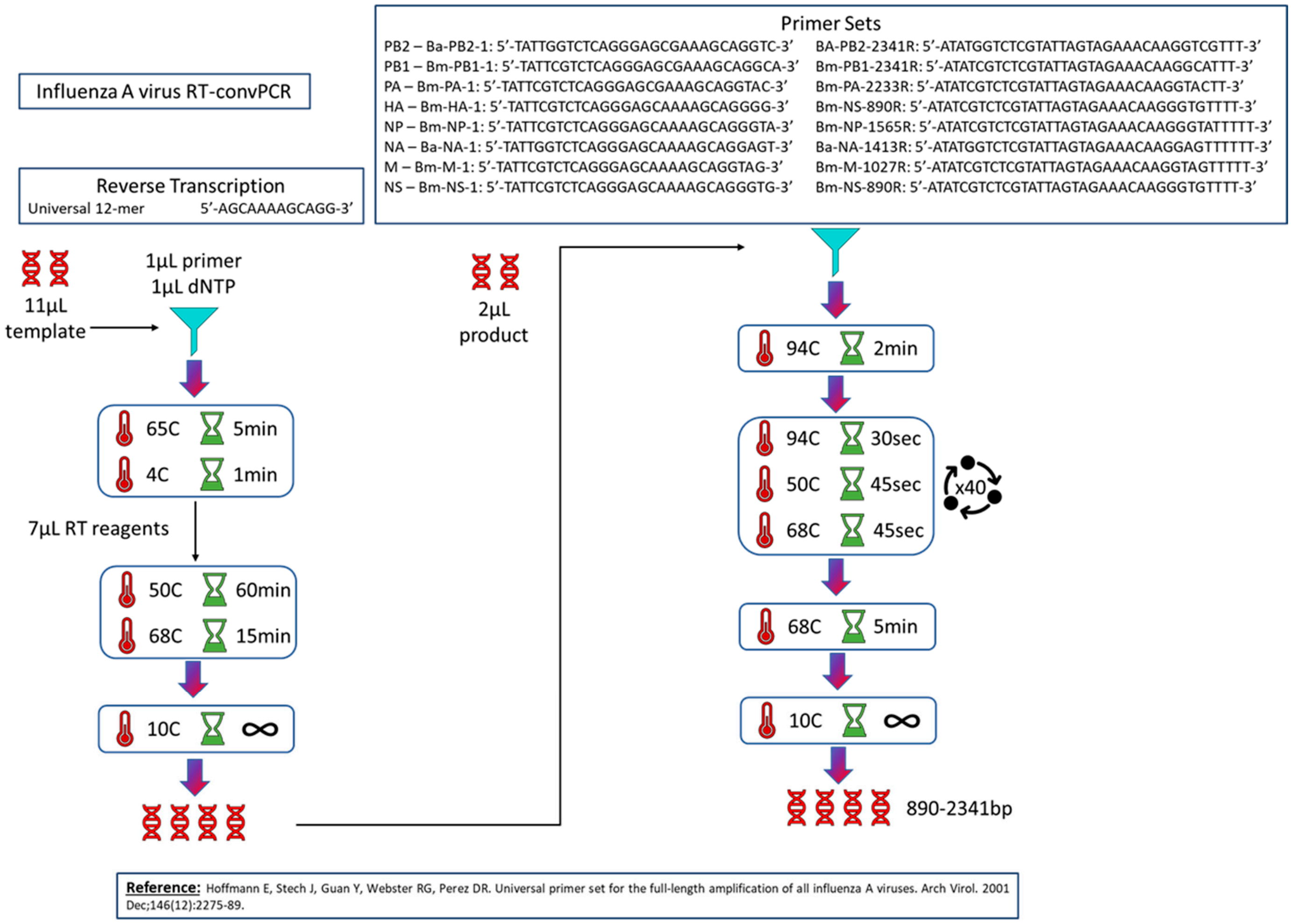

Figure S5. Detection of influenza A virus using reverse transcription (RT), conventional PCR (convPCR) against all 8 RNA gene segments [3]. The RT step utilizes a universal 12-mer primer. The primers specific to influenza A virus proteins for convPCR are listed. In the RT step, 1μL dNTPs (10mM) and 1μL RT primer (50μM) are mixed with 11μL extracted RNA. To this, 7μL of SuperScript III RT reagents (Invitrogen) are added. Finally, 2μL of the RT product is combined with 1μL forward/reverse primers (10μM) and Platinum Taq DNA Polymerase reagents (Invitrogen). The total volume of the PCR reaction is 50μL.

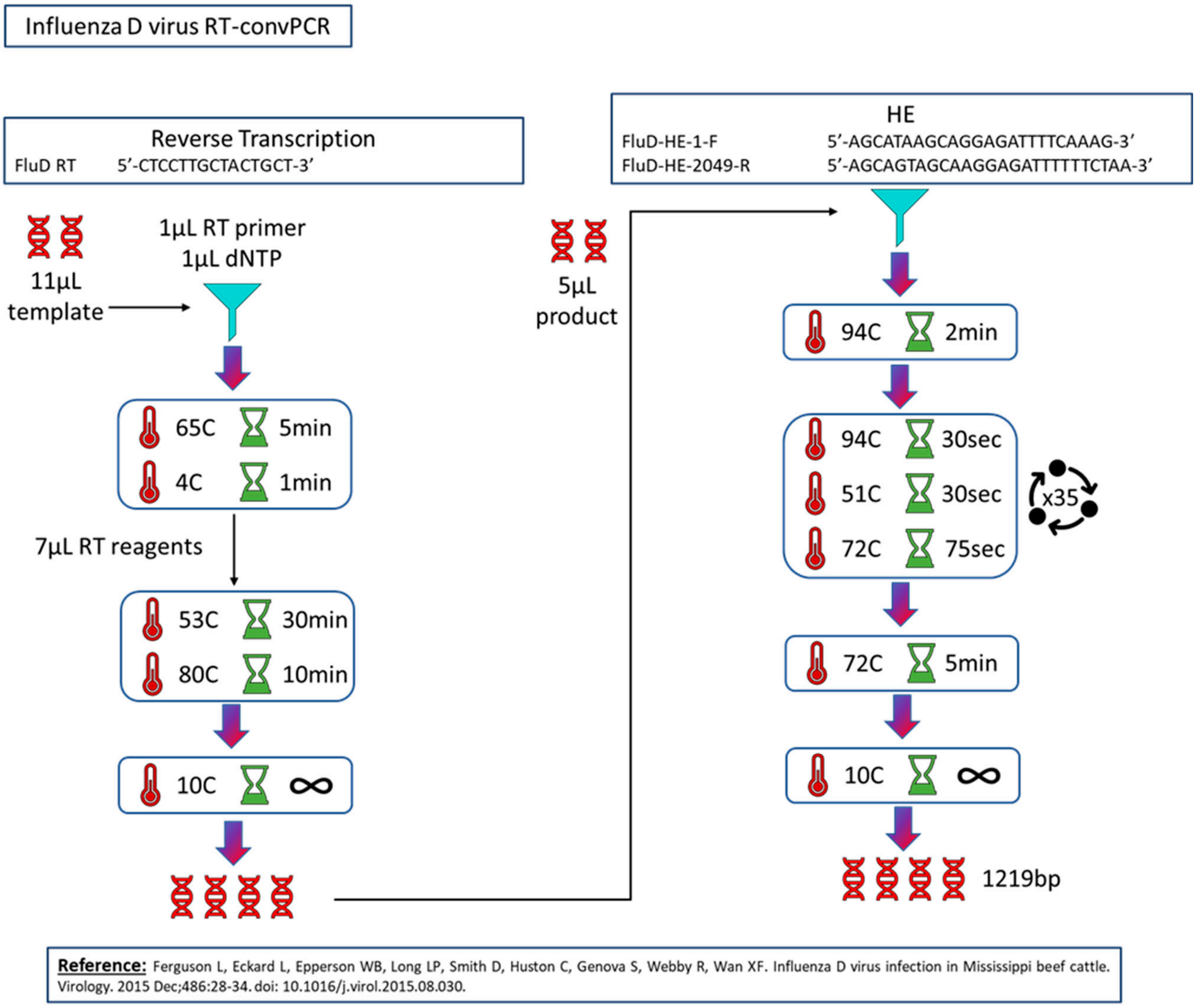

Figure S6. Detection of influenza D virus using reverse transcription (RT), conventional PCR (convPCR) [4]. The RT step utilizes a specific influenza D virus primer. The primers recognizing influenza D virus HE protein are listed. In the RT step, 1μL dNTPs (10mM), 1μL RT primer (2μM), and 2μL water are mixed with 11μL extracted RNA. To this, 7μL of SuperScript IV RT reagents (Invitrogen) are added. Finally, 5μL of the RT product is combined with 0.5μL forward/reverse primers (10μM) and Platinum Taq DNA Polymerase reagents (Invitrogen). The total volume of the reaction is 25μL.

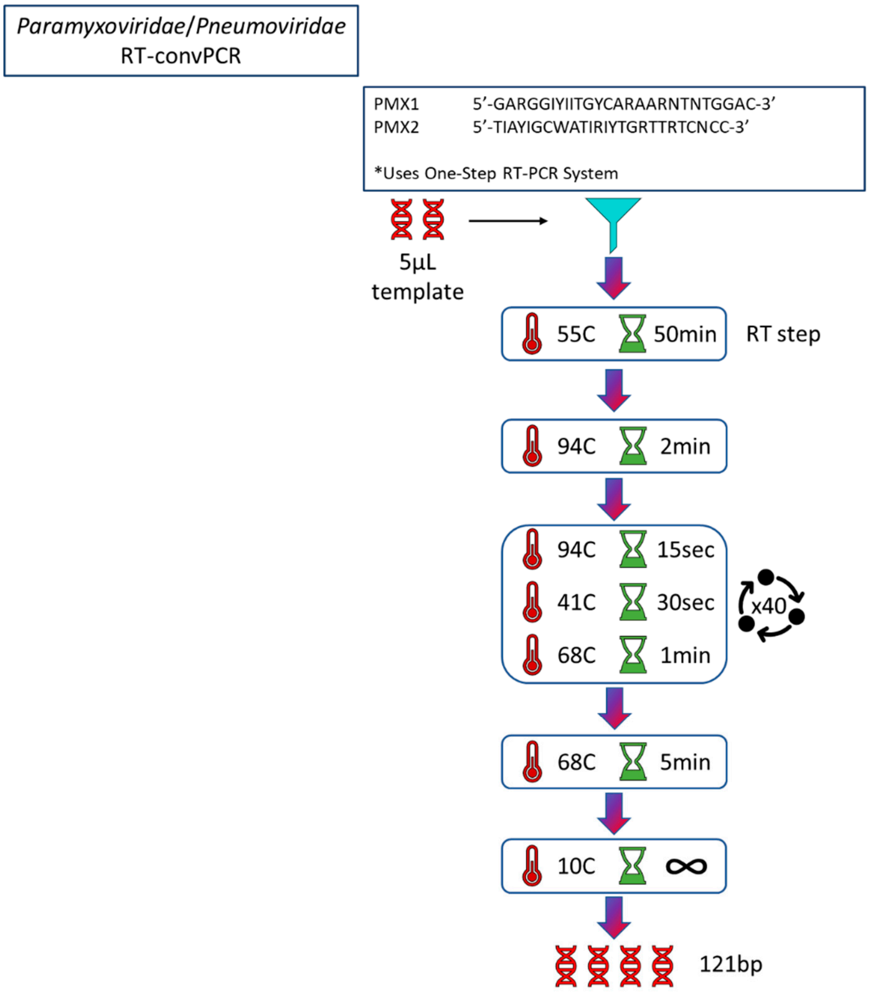

Reference: van Boheemen S, Bestebroer TM, Verhagen JH, et al. A family-wide RT-PCR assay for detection of paramyxoviruses and application to a large-scale surveillance study. PLoS One. 2012;7(4):e34961. doi:10.1371/journal.pone.0034961

Figure S7. Detection of *Paramyxoviridae*/*Pneumoviridae* viruses using reverse transcription (RT), conventional PCR (convPCR) [5]. The RT step utilizes the forward/reverse primers. The primers recognizing paramyxovirus are listed. This assay utilizes the SuperScript III One-Step RT-PCR System with Platinum Taq DNA Polymerase (Invitrogen). The RT-PCR reagents are combined with 1μL forward/reverse primers (10μM) and 5μL extracted RNA. The total volume of the reaction is 25μL.

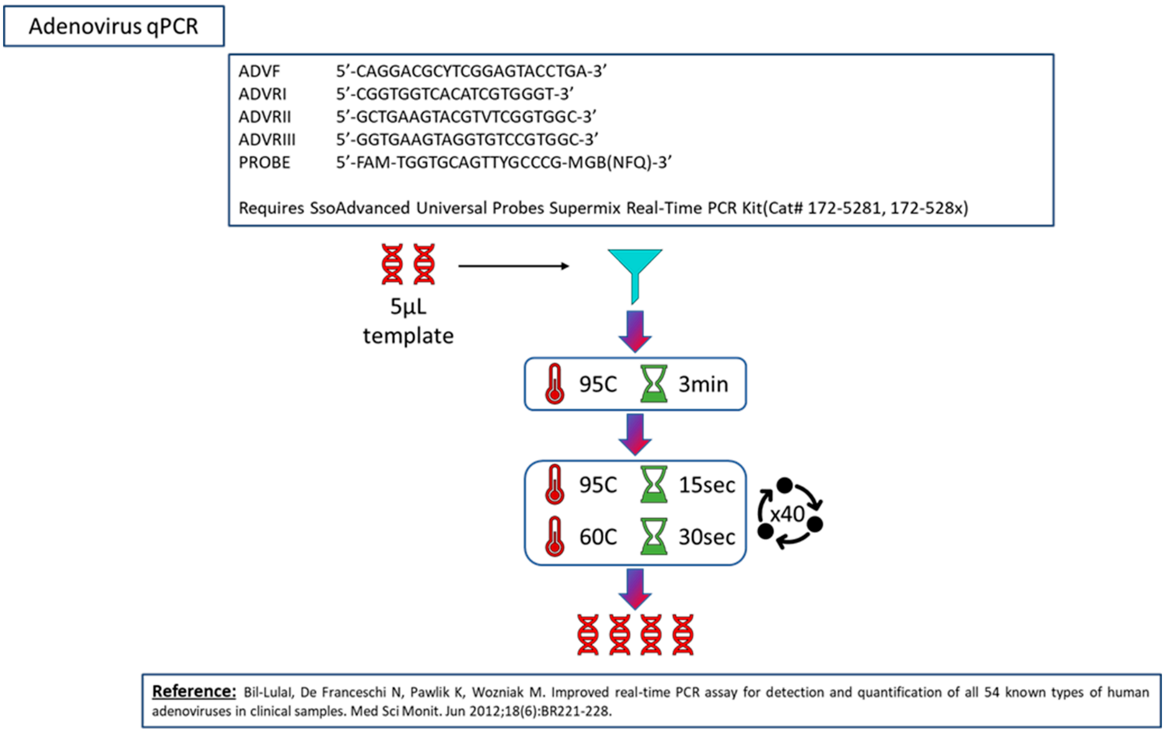

Figure S8. Detection of human adenoviruses using real-time PCR (qPCR) [6]. The primers and probe recognizing adenovirus are listed and include three reverse primers. This assay utilizes the SsoAdvanced Universal Probes Supermix Real-Time PCR kit (Bio-Rad Laboratories). To the supermix, 0.6µL forward primer (10µM), 0.7µL reverse primer I (25µM), 0.7µL reverse primer II (25µM), 0.6µL reverse primer III (10µM), 1µL probe (5µM), and 5µL extracted DNA are added. The total volume of the reaction is 20µL.

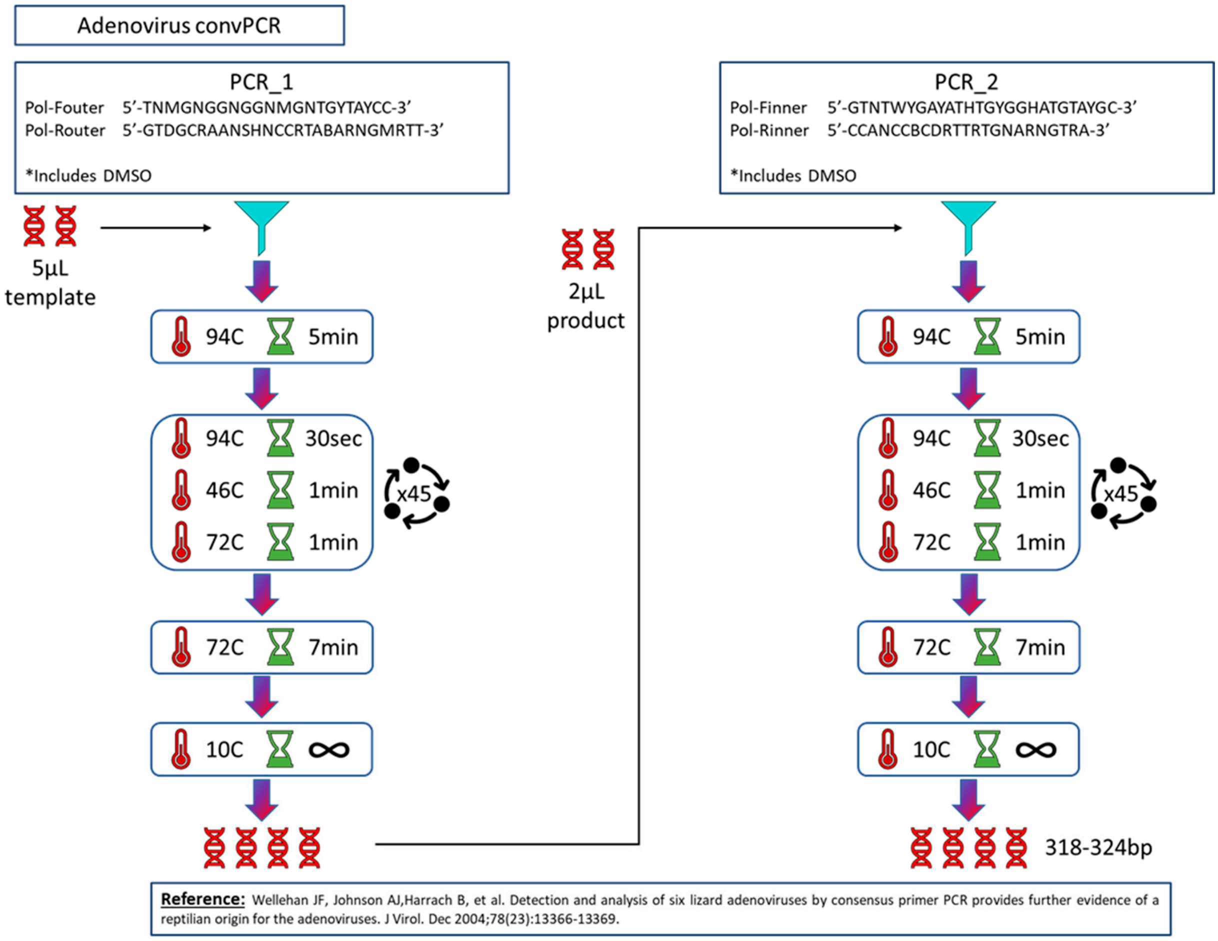

Figure S9. Detection of animal adenoviruses using conventional PCR (convPCR) [7]. The PCR 1 and PCR 2 primers recognizing adenovirus are listed. This assay utilizes the Platinum Taq DNA Polymerase kit (Invitrogen). For PCR 1, 0.5µL forward primer (25µM), 0.5µL reverse primer (25µM), 0.5µL dNTP (10mM), 0.24µL 100% dimethyl sulfoxide (DMSO), and 5µL extracted DNA are added to the PCR mix. The total volume of the reaction is 25µL. This is repeated for PCR 2 using the PCR 2 primers and 2µL of PCR 1 product.

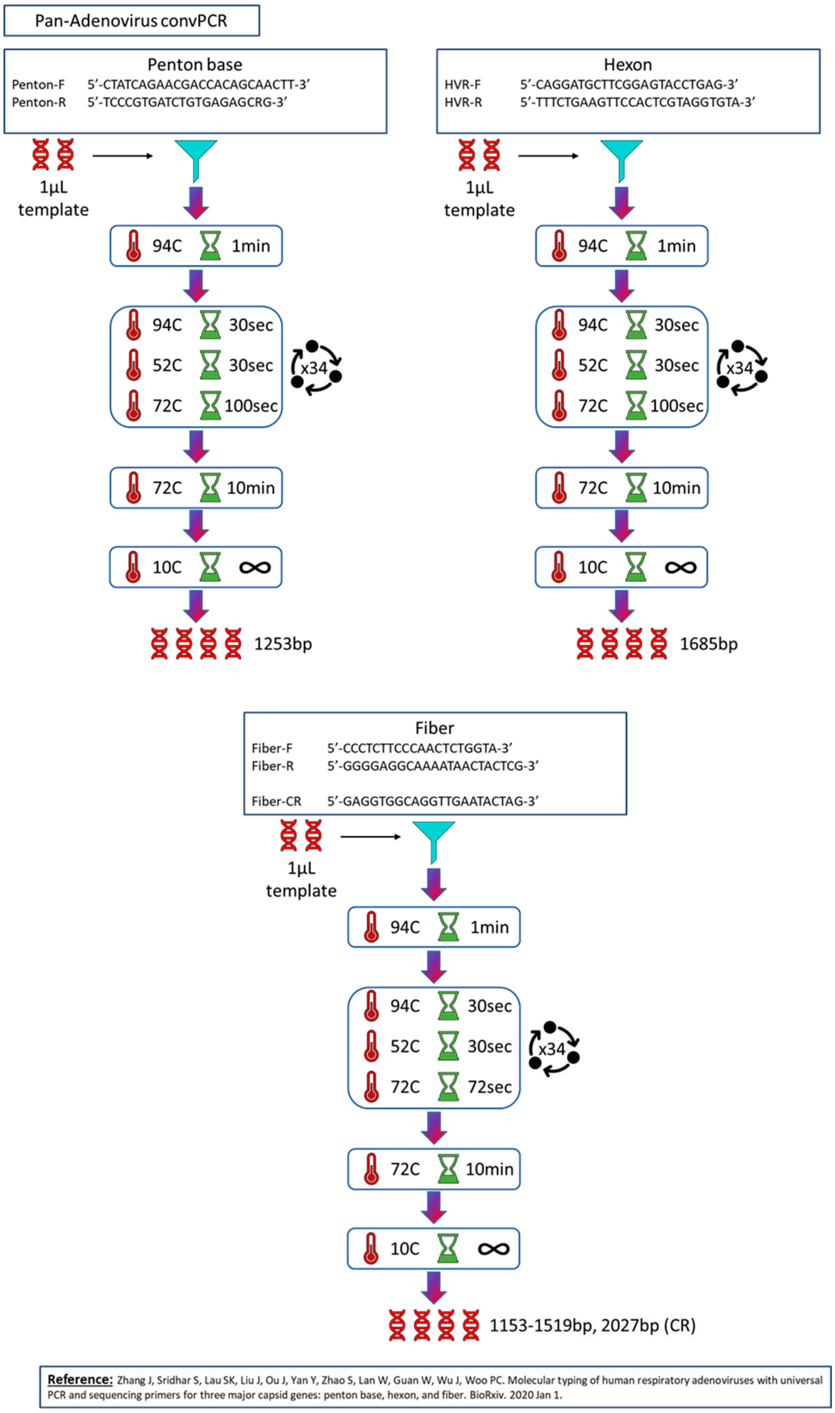

Figure S10. Detection of pan-adenoviruses using conventional PCR (convPCR) [8]. The primers recognizing adenovirus penton base, hexon, and fiber are listed. This assay utilizes the Platinum Taq DNA Polymerase kit (Invitrogen). For each reaction, 0.5μL forward primer (10μM), 0.5μL reverse primer (10μM), 0.5μL dNTP (10mM), and 1μL extracted DNA are added to the PCR mix. The total volume of the reaction is 25μL. Note the different PCR running conditions for the Fiber gene.

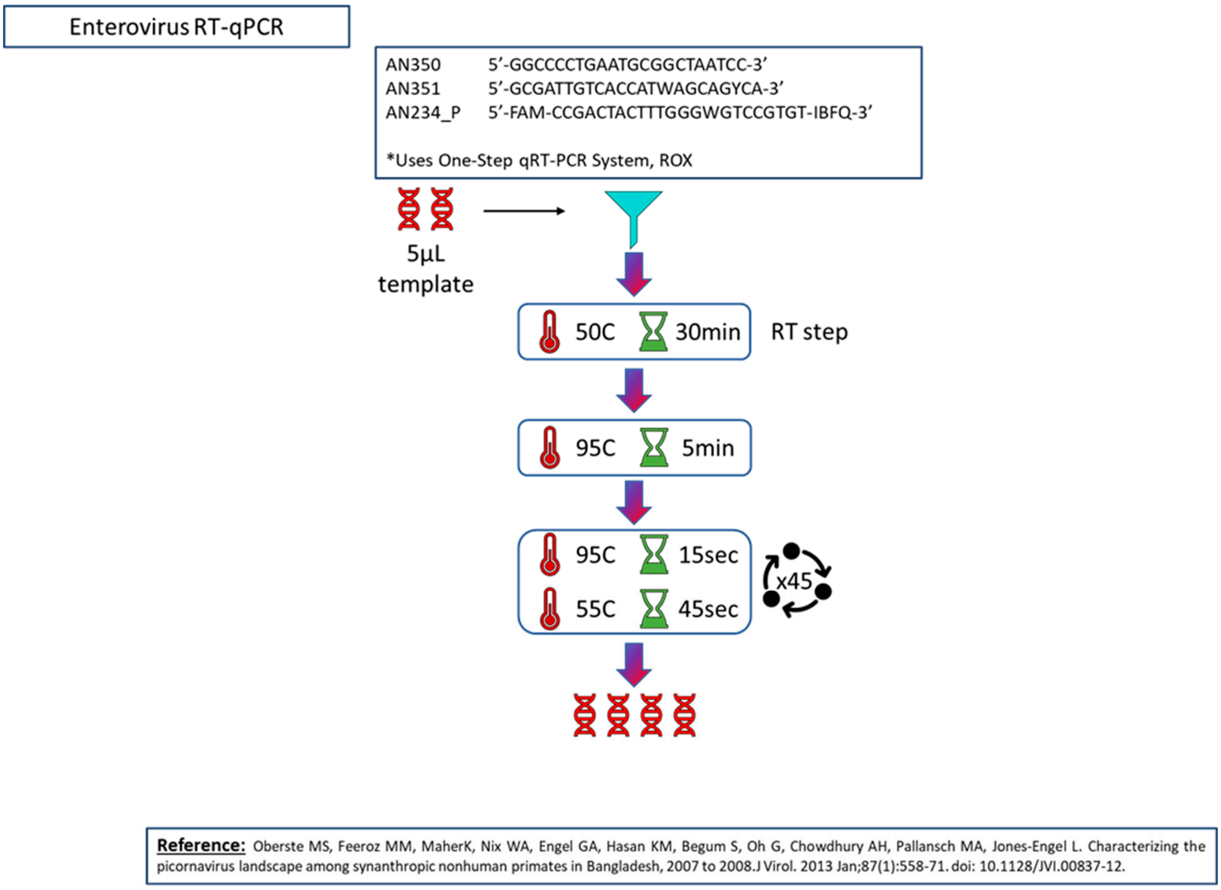

Figure S11. Detection of enterovirus using reverse transcription (RT), real-time PCR (qPCR) [9]. The primers and probe recognizing enterovirus are listed. This assay utilizes the SuperScript III Platinum One-Step qRT-PCR Kit (Invitrogen). To the qPCR mix, 0.2µL forward primer (40µM), 0.2µL reverse primer (40µM), 0.2µL probe (10µM), 0.4µL ROX (1:10 diultion), and 5µL extracted RNA are added. The total volume of the reaction is 20µL.

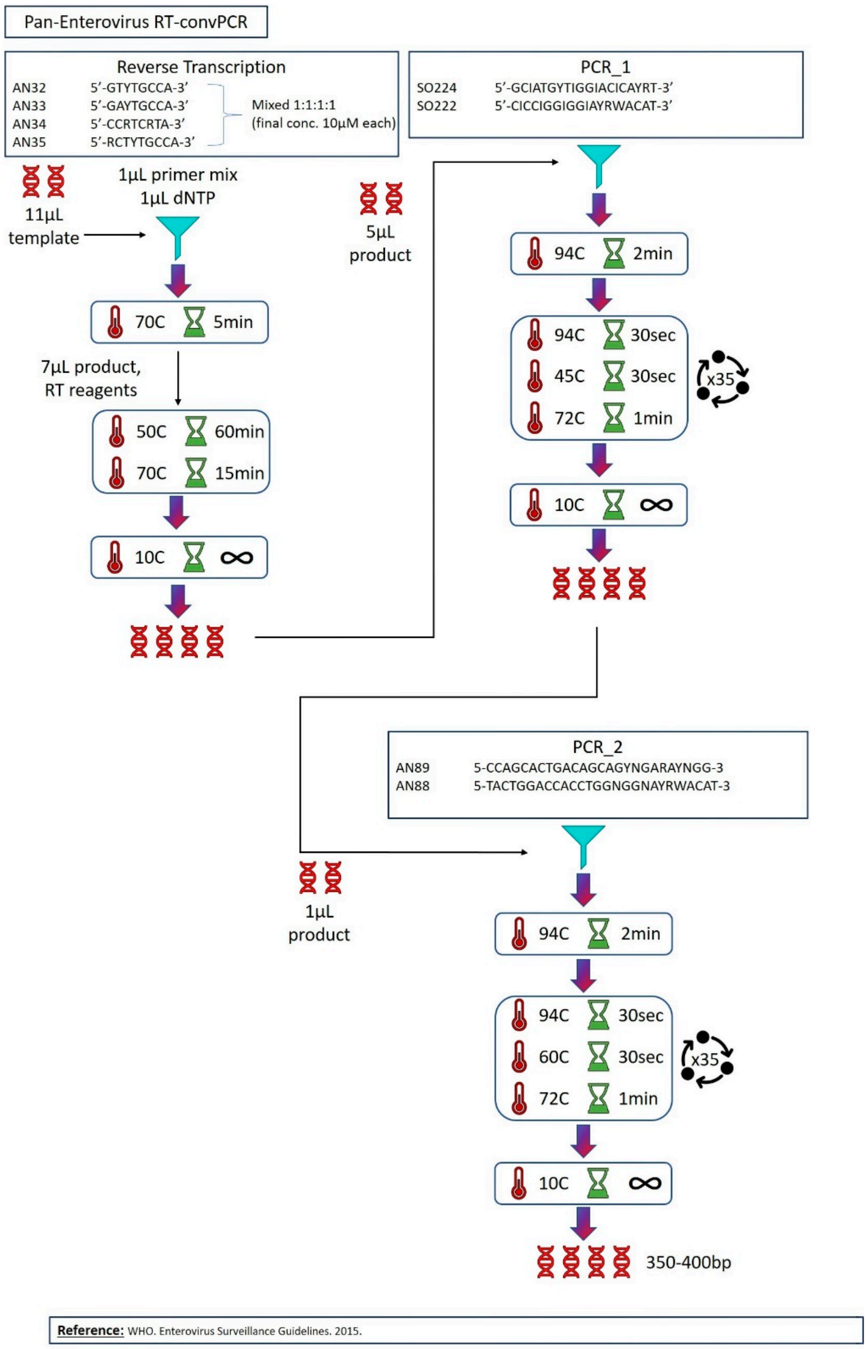

Figure S12. Detection of pan-enteroviruses using reverse transcription (RT), conventional PCR (convPCR) [10]. The RT step, PCR 1, and PCR 2 primers recognizing enterovirus are listed. This assay utilizes the SuperScript III RT Enzyme (Invitrogen) and Platinum Taq DNA Polymerase (Invitrogen) kits. For the RT step, the four RT primers (10µM) are combined in a 1:1:1:1 ratio. To 11µL extracted RNA, 1µL of the RT primer mix and 1µL dNTP (10mM) are added. Following incubation, 7µL of the RT reagents are added. For PCR 1, 0.5µL forward primer (10µM), 0.5µL reverse primer (10µM), 0.5µL dNTP (10mM), and 5µL DNA template are added to the PCR mix. The total volume of the reaction is 25µL. For PCR 2, 0.5µL forward primer (10µM), 0.5µL reverse primer (10µM), 0.5µL dNTP (10mM), and 1µL PCR 1 product are added to the PCR mix. The total volume of the reaction is 25µL.

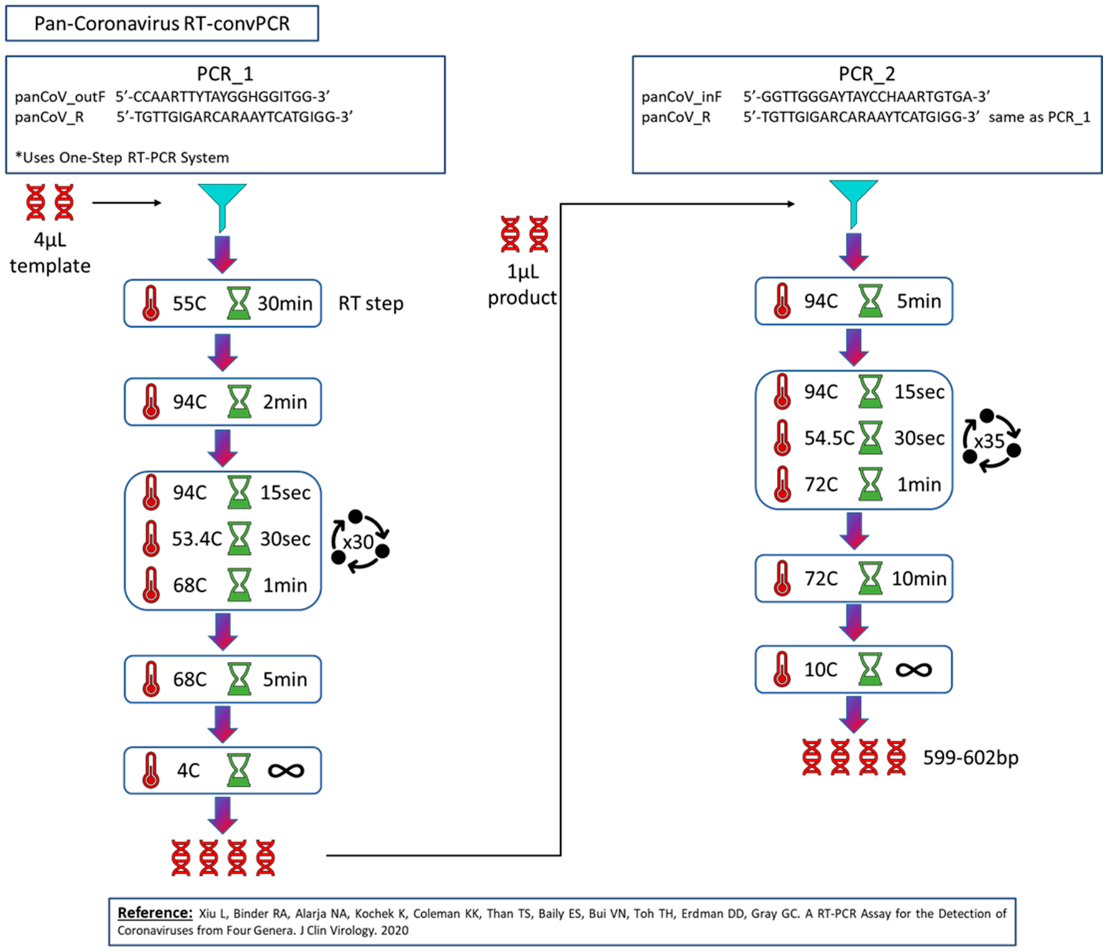

SFigure 13. Detection of pan-coronaviruses using reverse transcription (RT), conventional PCR (convPCR) [11]. The PCR 1 and PCR 2 primers recognizing coronavirus are listed. This assay utilizes the SuperScript III OneStep RT-PCR System with Platinum Taq DNA Polymerase kit (Invitrogen) in the first step and Platinum Taq DNA Polymerase kit (Invitrogen) in the second step. For PCR 1, 1μL of the forward primer (10μM), 1μL reverse primer (10μM), and 4μL extracted RNA are added to the PCR mix. The total volume of the reaction is 25μL. For PCR 2, 1μL forward primer (10μM), 1μL reverse primer (10μM), 0.5μL dNTP (10mM), and 1μL PCR 1 product are added to the PCR mix. The total volume of the reaction is 25μL.

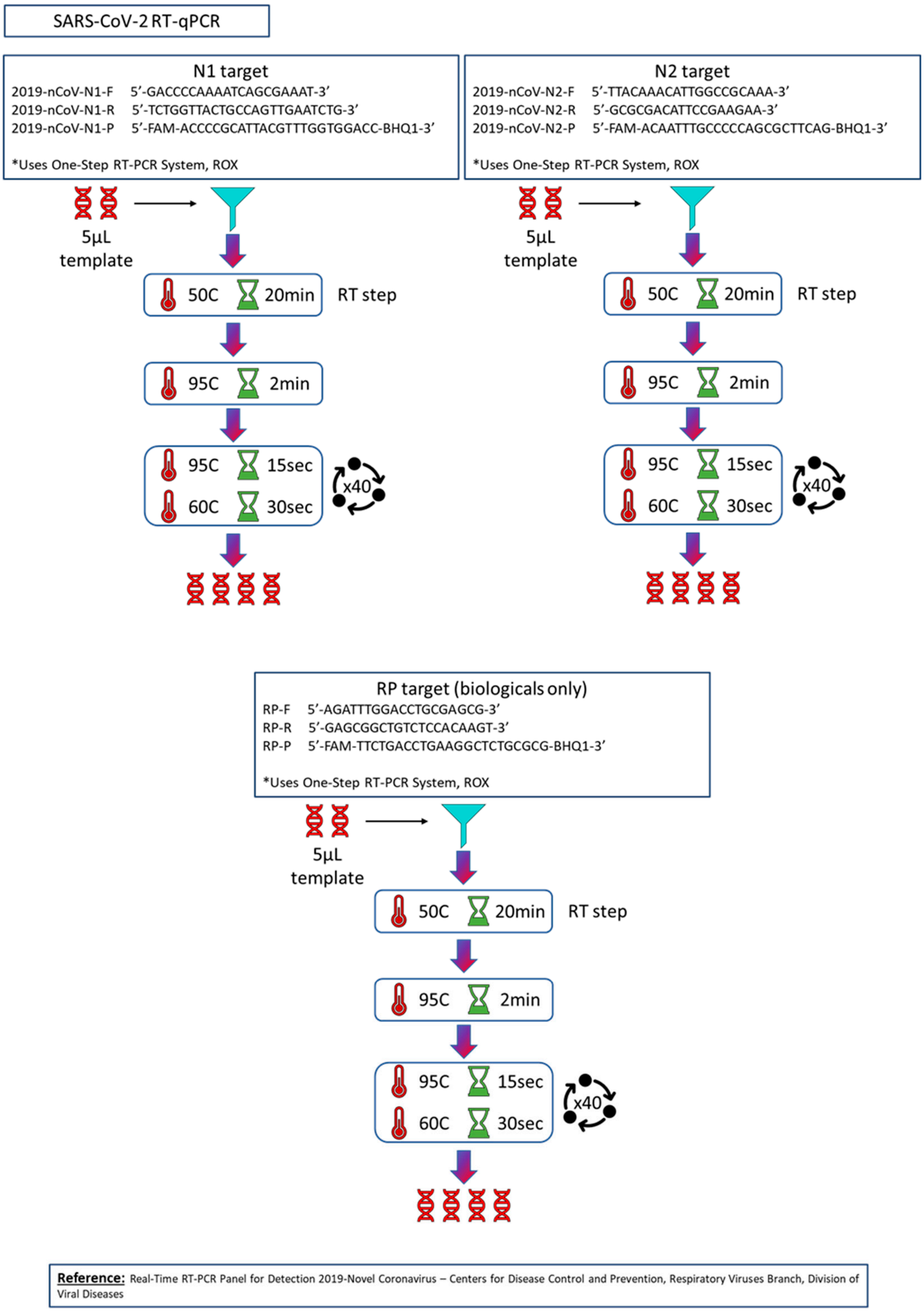

SFigure 14. Detection of SARS-CoV-2 using reverse transcription (RT), real-time PCR (qPCR) [12]. The primers and probe recognizing coronavirus N1 and N2 proteins, and RP control protein (only for human biological samples) are listed. These assays utilize the SuperScript III Platinum One-Step qRT-PCR Kit (Invitrogen) and 2019-nCoV CDC qPCR Probe Assay primer/probe mix (IDT). To the qPCR mix, 1.5μL N1/N2/RP primer/probe mix, 0.4μL ROX (1:10 diultion), and 5μL extracted RNA are added. The total volume of the reaction is 20μL.

References

1. Pabbaraju, K.; Wong, S.; Wong, A.; May-Hadford, J.; Tellier, R.; Fonseca, K., Detection of influenza C virus by a real-time RT-PCR assay. *Influenza Other Respir Viruses* **2013,** 7, (6), 954-60.
2. Hause, B. M.; Ducatez, M.; Collin, E. A.; Ran, Z.; Liu, R.; Sheng, Z.; Armien, A.; Kaplan, B.; Chakravarty, S.; Hoppe, A. D.; Webby, R. J.; Simonson, R. R.; Li, F., Isolation of a novel swine influenza virus from Oklahoma in 2011 which is distantly related to human influenza C viruses. *PLoS Pathog* **2013,** 9, (2), e1003176.
3. Hoffmann, E.; Stech, J.; Guan, Y.; Webster, R. G.; Perez, D. R., Universal primer set for the full-length amplification of all influenza A viruses. *Archives of Virology* **2001,** 146, 2275-2289.
4. Ferguson, L.; Eckard, L.; Epperson, W. B.; Long, L. P.; Smith, D.; Huston, C.; Genova, S.; Webby, R.; Wan, X. F., Influenza D virus infection in Mississippi beef cattle. *Virology* **2015,** 486, 28-34.
5. van Boheemen, S.; Bestebroer, T. M.; Verhagen, J. H.; Osterhaus, A. D.; Pas, S. D.; Herfst, S.; Fouchier, R. A., A family-wide RT-PCR assay for detection of paramyxoviruses and application to a large-scale surveillance study. *PLoS One* **2012,** 7, (4), e34961.
6. Bil-Lula, I.; De Franceschi, N.; Pawlik, K.; Mieczyslaw, W., Improved real-time PCR assay for detection and quantification of all 54 known types of human adenoviruses in clinical samples. *Medical Science Monitor* **2012,** 18, (6), 221-228.
7. Wellehan, J. F.; Johnson, A. J.; Harrach, B.; Benko, M.; Pessier, A. P.; Johnson, C. M.; Garner, M. M.; Childress, A.; Jacobson, E. R., Detection and analysis of six lizard adenoviruses by consensus primer PCR provides further evidence of a reptilian origin for the atadenoviruses. *J Virol* **2004,** 78, (23), 13366-9.
8. Zhang, J.; Sridhar, S.; Lau, S. K. P.; Liu, J.; Ou, J.; Yan, Y.; Zhao, S.; Lan, W.; Guan, W.; Wu, J.; Woo, P. C. Y.; Seto, D.; Zhang, Q., Molecular Typing of Human Respiratory Adenoviruses with Universal PCR and Sequencing Primers for Three Major Capsid Genes: Penton base, Hexon, and Fiber. *bioRxiv* **2020**.
9. Oberste, M. S.; Feeroz, M. M.; Maher, K.; Nix, W. A.; Engel, G. A.; Hasan, K. M.; Begum, S.; Oh, G.; Chowdhury, A. H.; Pallansch, M. A.; Jones-Engel, L., Characterizing the picornavirus landscape among synanthropic nonhuman primates in Bangladesh, 2007 to 2008. *J Virol* **2013,** 87, (1), 558-71.
10. World Health Organization; Centers for Disease Control and Prevention Enterovirus Surveillance Guidelines. https://www.euro.who.int/__data/assets/pdf_file/0020/272810/EnterovirusSurveillanceGuidelines.pdf
11. Xiu, L.; Binder, R. A.; Alarja, N. A.; Kochek, K.; Coleman, K. K.; Than, S. T.; Bailey, E. S.; Bui, V. N.; Toh, T. H.; Erdman, D. D.; Gray, G. C., A RT-PCR assay for the detection of coronaviruses from four genera. *J Clin Virol* **2020,** 128, 104391.
12. Centers for Disease Control and Prevention *CDC 2019-Novel Coronavirus (2019-nCoV) Real-Time RT-PCR Diagnostic Panel*; Centers for Disease Control and Prevention, Division of Viral Diseases: 2020.
